# Morphologic, cytometric, quantitative transcriptomic and functional characterisation provide insights into the haemocyte immune responses of Pacific abalone (*Haliotis discus hannai*)

**DOI:** 10.3389/fimmu.2024.1376911

**Published:** 2024-07-02

**Authors:** Zeyuan Ma, Yunlong Wu, Yu Zhang, Weini Zhang, Mingmei Jiang, Xiaoyue Shen, Hailian Wu, Xinhua Chen, Guilan Di

**Affiliations:** ^1^ State Key Laboratory of Mariculture Breeding, Key Laboratory of Marine Biotechnology of Fujian Province, Institute of Oceanology, College of Marine Sciences, Fujian Agriculture and Forestry University, Fuzhou, China; ^2^ Laboratory for Marine Biology and Biotechnology, Qingdao National Laboratory for Marine Science and Technology, Qingdao, China

**Keywords:** *Haliotis discus hannai*, transcriptomic, haemocyte, phagocytosis, electron microscopy

## Abstract

In recent years, the abalone aquaculture industry has been threatened by the bacterial pathogens. The immune responses mechanisms underlying the phagocytosis of haemocytes remain unclear in *Haliotis discus hannai*. It is necessary to investigate the immune mechanism in response to these bacterial pathogens challenges. In this study, the phagocytic activities of haemocytes in *H. discus hannai* were examined by flow cytometry combined with electron microscopy and transcriptomic analyses. The results of *Vibrio parahaemolyticus*, *Vibrio alginolyticus* and *Staphylococcus aureu* challenge using electron microscopy showed a process during phagosome formation in haemocytes. The phagocytic rate (PP) of *S. aureus* was higher than the other five foreign particles, which was about 63%. The PP of *Vibrio harveyi* was about 43%, the PP peak of *V. alginolyticus* in haemocyte was 63.7% at 1.5 h. After *V. parahaemolyticus* and *V. alginolyticus* challenge, acid phosphatase, alkaline phosphatase, total superoxide dismutase, lysozyme, total antioxidant capacity, catalase, nitric oxide synthase and glutathione peroxidase activities in haemocytes were measured at different times, differentially expressed genes (DEGs) were identified by quantitative transcriptomic analysis. The identified DEGs after *V. parahaemolyticus* challenge included haemagglutinin/amebocyte aggregation factor-like, supervillin-like isoform X4, calmodulin-like and kyphoscoliosis peptidase-like; the identified DEGs after *V. alginolyticus* challenge included interleukin-6 receptor subunit beta-like, protein turtle homolog B-like, rho GTPase-activating protein 6-like isoform X2, leukocyte surface antigen CD53-like, calponin-1-like, calmodulin-like, troponin C, troponin I-like isoform X4, troponin T-like isoform X18, tumor necrosis factor ligand superfamily member 10-like, rho-related protein racA-like and haemagglutinin/amebocyte aggregation factor-like. Some immune-related KEGG pathways were significantly up-regulated or down-regulated after challenge, including thyroid hormone synthesis, Th17 cell differentiation signalling pathway, focal adhesion, melanogenesis, leukocyte transendothelial migration, inflammatory mediator regulation of TRP channels, ras signalling pathway, rap1 signalling pathway. This study is the first step towards understanding the *H. discus hannai* immune system by adapting several tools to gastropods and providing a first detailed morpho-functional study of their haemocytes.

## 1 Introduction

An organism’s immune system is essential to its survival (1). Innate immunity and acquired immunity are components of immune systems. Innate immunity, on the other hand, offers immediate defence against infection as opposed to acquired immunity’s durability (2). Since they lack traditional acquired immunity, invertebrates are assumed to primarily rely on innate immunity to fight against invasive infections and adapt to environmental challenges (3, 4).

According to Pham et al. ‘s study (5), phagocytes are essential for maintaining immunological homeostasis and killing pathogens. Phagocytes participate in phagocytosis, oxidative killing and encapsulation processes. Both vertebrate and invertebrate blood cells have an essential role in phagocytosis, which is connected to pathogen defence and immunological surveillance (6). Phagocytes, with their capacity to engulf and eliminate microbial pathogens, are probably going to play a major role in immunological defence, particularly in invertebrates (4). According to recent investigations, phagocytes in mollusks have been seen to capture and engulf invasive bacteria (7). For instance, the lysosomal proteolytic pathway was used by *Euprymna scolope*s’ phagocytes to break down germs that have been ingested (8). These findings emphasise the regulation of phagocytic activity in innate immunity (9).

Mollusc haemocytes are known to perform many functions, including phagocytosis, killing invasive microbes, identifying foreign particles and oxidatively eliminating them (10). Haemocytes have been demonstrated in earlier research to be crucial for mollusk immunological responses in both humoral and cellular immunity. Molluscan haemocytes play a crucial role in phagocytosis. In the blue mussel *Mytilus edulis*, for example, phagocytosis has been shown to significantly decrease after cadmium exposure, and several immune-related genes linked to phagocytosis in *M. edulis* were significantly up-regulated after *Vibrio splendidus* challenge (11).

Mammals’ phagocytic killing mechanisms have been extensively investigated (12), but these mechanisms in gastropods are still not well understood. Phagocytosis in gastropods has been documented in the caenogastropod *Viviparus*, the pulmonate gastropods *Lymnaea stagnailis*, *Biomphalaria glabrata*, *Cerithidea californica*, the marine gastropods *H. discus hannai*, *Haliotis asinine*, *Haliotis tuberculata*, *Littorina littorea*, and ivory shell *Babylonia areolata* (13, 14). Nevertheless, the majority of research has focused on the morphological traits and immune capabilities of haemocytes; hence, functional assessments and characterisation of phagocytosis in gastropod haemocytes are still missing.

The coastal regions of Liaoning, Jiangsu, Shandong and northern Japan are the primary distribution areas for the Pacific abalone *H. discus hannai*. China’s mariculture of *H. discus hannai* has grown significantly in the last several years, and has become one of the important mariculture shellfish (15). In addition, it is referred to as marine soft gold and is more valuable commercially and culturally than *H. tuberculata* and *Haliotis diversicolor*. Despite the fact that abalone aquaculture is a massive industry in China, there are still issues. One of these is the decrease in abalone germplasm caused by repeated inbreeding. In recent years, there have been many disease outbreaks in China’s abalone industry, which have caused significant financial losses (16). Little is known about the innate defence of *H. discus hannai* haemocytes. It is critical to understand *H. discus hannai*’s immune system in order to reduce disease-associated mortality, which makes it significant for *H. discus hannai* farming.

In marine environments, bacterial pathogens are ubiquitous and cause continuously threaten to the health of the majority of aquaculture species. Particularly, the vibriosis disease is a significant challenge to the culture of mollusks, as many *Vibrio* species are thought to be the cause of widespread outbreaks of abalone mortality. One of principal bacterial pathogen is *V. parahaemolyticus* in *H. discus hannai*, it is known to cause “withering syndrome” and “outer velum breaking disease” in the abalone aquaculture industry, which can result in significant losses (17, 18). For instance, significant stock losses of *Haliotis diversicolor supertexta* were attributed to *V. parahaemolyticus* in China (19). One kind of conditional pathogen found in oceans is *V. alginolyticus* (20). When the environment deteriorates or the immune function decreases, the disease is more likely to spread (21). While *Vibrio splendidus* was isolated from moribund *Haliotis rubra* and *Haliotis laevigata* during disease outbreaks in Australia (22), infections of *Vibrio carchariae* and *V. harveyi* have been reported in *H. tuberculata* along the French coast (23) and in *H. discus hannai* in Japan (24), respectively.

The occurrence of several illnesses has hampered the cultivation of *H. discus hannai* due to a lack of studies on innate defence of *H. discus hannai* haemocytes and disease control. “Omics” research has been more productive and economical in the last several years. For the purpose of identifying genes linked to immunity and studying the phagocytic killing mechanisms underlying mollusk immunity, transcriptomics can be a very useful tool. Even so, the abalone genome’s initial draft has already been made public. Currently, inadequate sequence splicing quality and a dearth of pertinent gene resource data are impeding research (25). This work used transcriptomics, flow cytometry and histological characteristics to shed light on the immune responses by haemocytes in *H. discus hannai*. Quantitative transcriptome studies of the haemocytes were carried out following the *V. parahaemolyticus* and *V. alginolyticus* challenge in order to determine the immunological pathways linked to the phagocytosis by the haemocytes. Investigating the immunological response of the *H. discus hannai* haemocytes to foreign pathogens and fluorescent polystyrene beads, as well as examining the fates of the bacteria following ingestion, were the objectives of this work. Numerous substantially differentially expressed genes (DEGs) were found to be engaged in signalling networks relevant to the immune system.

## 2 Materials and methods

### 2.1 Animal rearing and manipulation

Adult Pacific abalones (body length 5.80 ± 0.60 cm, body weight 17.65 ± 3.50 g; n = 500) were collected from Jinjiang Fuda Abalone Fisheries Co. Ltd (Jinjiang, Fujian, China) and acclimated for 14 days before the experiment under the same cultivation conditions (24 ± 1°C, 33‰ salinity, and 7.8 pH). Then, the abalones were kept in a recirculating system with a sand- filter, where they were fed red alga *Gracilaria lemaneiformis* or sea tangle (*Laminaria japonica*) once a day, and saltwater was changed daily. The system was kept at consistent temperatures (23°C) and dissolved oxygen levels (6.2 mg/L). Every day, seawater was added to the aquariums, and all animal studies were carried out in accordance with the ‘Guidelines for Experimental Animals’ of the Ministry of Science and Technology (Beijing, China; No. [2006] 398, 30 September 2006). All of the experiments involving animals reported in this study were approved by the Institutional Animal Care and Use Committee (IACUC) of Fujian Agriculture and Forestry University.

### 2.2 Haemocyte preparation

The abalones were cleaned using gauze and absorbent cotton before being sliced lengthwise in the center of the foot muscle using a sterilised scalpel. Every individual was collected about 2.0–3.0 mL of hemolymph, which was then divided into individual 15 mL centrifuge tubes. Hemolymph samples immediately mixed with same volume modified Alsever’s solution MAS (the MAS anticoagulant contained 20.8 g L^−1^ of glucose; 3.36 g L^−1^ of EDTA; 8 g L^−1^ of sodium citrate and 22.5 g L−1 of NaCl.) to prevent aggregation, as recommended. After centrifuging the hemolymph for five minutes at 700 g to extract circulating haemocytes and supernatant for subsequent experiment.

### 2.3 Morphologic observation on haemocytes

A total of three cleaned microscope slides were filled with 50 μL of hemolymph solution per sample. One was utilised for direct light microscope observation, and the other was employed to prepare smears. Haematoxylin-eosin (HE) staining and Fast Wright-Giemsa staining were applied to two smear preparations per sample. All the samples were observed using a Leica DM 4000 B microscope and Leica DFC 425 C image sensor. Each type of haemocyte was counted, 100 cells per abalone were measured, there were 6 abalones for cell counts and size measurement.

### 2.4 Bacterial strains and preparation of bacterial suspensions

Experiments were carried out using the Gram-positive bacterium *S. aureus* and the Gram-negative bacterium *Escherichia coli*, which were obtained from Hope Bio-Technology Co., Ltd., Qingdao, Shandong province, China. Bacterium were activated according to the supplier’s guidelines. Take a small amount of *E. coli*, *S. aureus* separately and inoculate them on Luria-Bertani (LB) solid culture medium with inoculation rings. After culturing at 37°C for 16–18 hours, select two monoclonal strains of bacteria and inoculate them onto LB liquid culture medium. Shake and incubate at 37°C for 16–18 hours. Bacteria were collected by centrifugation at 3 000 g for 10 min at 4°C and resuspension in 0.85% sterile saline solution rinsed twice. The bacterial concentration in this investigation was around 10^9^ colony forming unit (CFU)/ml. Adjust the bacterial suspension to the required bacterial concentration for the experiment using phosphate buffer.

*V. harveyi, V. alginolyticus* and *V. parahaemolyticus* (isolated from diseased Pacific abalones and kept in our laboratory) were employed in the bacterial challenge experiment where the frozen culture was revived from -70°C storage. *V. harveyi, V. alginolyticus* and *V. parahaemolyticus* were cultivated overnight at 37°C in LB medium (containing 10 g/L peptone, 5 g/L yeast extract, and 10 g/L NaCl) and extracted via centrifugation (4 000 g, 10 min, 4°C). The pellet was then washed three times in sterile phosphate-buffered saline (PBS) with a pH of 7.4 before being resuspended in 0.9% normal saline (NS). The concentration of *V. parahaemolyticus* (1.0 × 10^8^ CFU/mL) was determined by analysing the findings of the pre-experimental stage. Haemocytes were treated with Vibrio for 1.5 hours at 28°C at a haemocyte/bacteria ratio of 1:10.

### 2.5 Preparation of haemocytes challenged by *V. alginolyticus* and *V. parahaemolyticus*

The bacterial challenge employed two species of Vibrios, *V. alginolyticus* and *V. parahaemolyticus*, which were previously isolated from moribund abalone and shown to be pathogenic to abalone (26, 27). A total volume of 50 μL *V. alginolyticus* (or *V. parahaemolyticus*) was injected into the abalone foot of 180 *H. discus hannai* specimens. 180 abalones which did not receive the bacterial challenge received an injection of sterile premixed phosphate buffered solution (PBS) and were used as the C group. Another 180 abalones were used as the “P” group and were given an injection of *V. parahaemolyticus* suspension at a concentration of 1.0 ×10^7^ CFU/mL (50 μL) (which had been demonstrated to be the sub-lethal concentration in our previous unpublished experiment) in a PBS solution into the pedal sinus using a 100 μL syringe. As the “A” group, another 180 abalones were given an injection of *V. alginolyticus* slurry at a concentration of 1.0 ×10^7^ CFU/mL (50 μL). After that, the treated animals were raised separately from the 180 control animals. Abalones from the C, P, and A groups had their hemolymph collected at 0, 6, 12, 24, 48, and 72 hours after the injection (n= 6).

The hemolymph samples were transferred to 1.5 mL Eppendorf tubes. All hemolymph solutions containing anticoagulant, the hemolymph was centrifuged at 700 g to separate the haemocytes and supernatant, the haemocytes were preserved at −80°C for subsequent transcriptomic analysis. The supernatant was preserved at −80°C for subsequent measure the average protein concentration, activation of acid phosphatase (ACP), alkaline protease (AKP), total antioxidant capacity (T-AOC), total superoxide dismutase (T-SOD), lysozyme (LZM), nitric oxide synthase (NOS), glutathione peroxidase (GSH-Px) and catalase (CAT).

### 2.6 Preparation of electron microscopy samples

For transmission electron microscopy (TEM), haemocytes were incubated for 1.5 h at 28°C with *V. parahaemolyticus* or *V. alginolyticus* or *S. aureus* at a bacteria/haemocyte ratio of 10:1. Then, haemocytes were fixed in PBS with 2.5% glutaraldehyde for 3 hours at 4°C (pH 7.4). Following fixation, the haemocytes were washed three times in 0.1 mol/L PBS (pH 7.4) before being transferred to 1% osmium tetraoxide at 4°C for two hours. Haemocytes post bacterial challenge were subsequently washed in PBS. Haemocyte pellets were generated in 2% agarose when the remaining glutaraldehyde and osmium tetraoxide were removed. Following immersion in propylene oxide, a dehydration procedure (50%, 70%, 80%, 90%, 95%, and 100% ethanol) was carried out. Finally, the fixed samples were embedded in Epon 812 resin (TAAB, UK) and cut into 60-nm thick ultrathin slices with an ultramicrotome (Leica CM1520). TEM was used to investigate ultrathin slices (60 nm) stained with 2% uranyl acetate and 1% lead citrate. A Hitachi TEM equipment (Model H-7650) was used to view and photograph the sections.

### 2.7 Determination of phagocytosis by haemocytes using flow cytometry

Flow cytometry was carried out using a FACSCalibur (Becton-Dickinson Immunocytometry Systems, San Jose, CA) coupled with a single argon ion laser with 488 nm filtered emission. Fluorescence photomultiplier bandpass filters were 530 nm (green fluorescence, FL1) and 585 nm (yellow/orange fluorescence, FL2). Side scatter (SSC) and fluorescence data were recorded on a log scale, whereas forward scatter (FSC) data was obtained on a linear scale. Each haemocyte sample had 10,000 events recorded. To establish logical zones and colour gating analyses of fluorescence data, the Cell Quest^®^ software (Becton-Dickinson, San Jose, CA, USA) was employed.

Total circulating haemocytes count (THC): SYBR Green I (Sigma-Aldrich, USA) was used for THC detection. A fluorescent dye that binds to double-stranded DNA. In brief, 200 μl of fresh hemolymph was fixed in an equivalent volume of 3% formalin solution. Fixed circulating haemocytes were treated for 120 minutes in the dark at room temperature with 1,000 x SYBR green I before being run in a flow cytometer. The number of undamaged cells was counted in 1 minute using a flow cytometer. The quantity of cells ml^−1^ hemolymph is used to report THC. The cytometer’s flow rate was determined using the methods previously mentioned (28). The quantity of cells ml^−1^ hemolymph is used to report THC.

*Ex vivo* phagocytosis was quantified by measuring the ability of haemocytes to ingest fluorescent beads. Samples containing 500 μL of anticoagulant MAS were mixed with 500 μL of haemocytes (about 2.16 × 10^6^ cells/mL). Next, 500 μL of haemocytes was mixed with 10 μL of a 1/10 dilution of fluorosphere microspheres with a diameter of 1 μm (FluoSpheresTM carboxylate, 1 μm, red, Life Technologies Corporation) and allowed to incubate at room temperature in the dark. The final ratio of beads to haemocytes was 100/1, while the final concentration of fluorescent beads was about 10^8^ mL^-1^. It is simple to discriminate between the endogenous baseline fluorescence of non-phagocytic haemocytes -containing beads and that of haemocytes-containing one, two, three, or more beads. Also, it is simple to determine how many beads each cell phagocytizes. Phagocytosis capacity was defined by Hégaret et al. (29) as the proportion of haemocytes-engulfed beads above three. In our investigation, we used this methodology. A fluorescence plot was used to calculate the proportion of cells that contained fluorescent beads. Haemocytes were conducted 0.5 h, 1.0 h, 1.5 h, 2.0 h, 2.5 h post-challenge by fluorescent beads on three randomly selected abalone from each of the tanks.

Fluorescein Isothiocyanate (FITC) -labelling of microorganisms was performed follows: *E. coli* and *S. aureus* were inoculated on LB at 37°C for 16–18 hours. Centrifugation was used to collected *V. harveyi, V. alginolyticus* and *V. parahaemolyticus*, which was cultured in LB medium at 28°C for the whole night. The optical densitometric approach was used to quantify the concentration of bacteria, and quantitative plate counting was used to confirm the results. The bacteria were mixed with fluorescein isothiocyanate (FITC, 1 mg/mL, Sigma-Aldrich, USA) after bacteria were washed three times with PBS (pH 7.4) at room temperature. Following that, three PBS washes (pH 7.4) were performed on the FITC-labelled microorganisms. Haemocytes were resuspended in MAS. A blood counting chamber and fluorescence photometer were used to count the number of haemocytes-positive cells and FITC-labelled bacteria in order to guarantee that the bacteria included in the study ultimately had the same FITC concentration. There were more than 95% FITC-positive bacteria present. By using quantitative plate counting, the percentage of bacterial viability in an aliquot of each population was found to be 96%–98%. At a ratio of 10 bacteria per haemocyte, FITC-labelled bacteria (about 2 × 10^7^ CFUs) were introduced to haemocyte suspensions (roughly 1.8 × 10^6^ cells), and the mixture was incubated for 0.5 h, 1.0 h, 1.5 h, 2.0 h, 2.5 h at 28°C. A negative control was an unintubated tube with only haemocytes. After centrifuging the suspensions to extract the haemocytes, extra microorganisms were eliminated by repeating three PBS washes. After being fixed for 25 minutes in 4% paraformaldehyde, haemocytes were examined using flow cytometry. The proportion of FITC-positive haemocytes was used to calculate the phagocytosis rate. The boundaries of positive and negative fluorescence were confirmed by processing FITC-stained and unstained bacteria phagocytosis mixtures. The FITC-positive and -negative cells were then gated and sorted according to the intensity of their FITC fluorescence. The dot plots were used to identify the gating zone, which removed dead cells. A total of 20,000 haemocytes units were processed for every sample. Green fluorescence (FL-1 detector) was used to measure the phagocytosis of the haemocytes linked with fluorescent beads. It is possible to precisely assess phagocytic activity analytically to calculate the mean fluorescence of cells, indicated in this instance by the proportion of cells that fluoresce more than three beads. The fluorescence signal indicated the phagocytosis area. The percentage of haemocytes that consumed three or more fluorescent beads was used to calculate the phagocytic activity of haemocytes (30). Three runs of this experiment were conducted.

### 2.8 Assessment of enzymatic activities

Enzymatic activity assays were carried out according to the manufacturer’s instructions using a standard assay kit from the Nanjing Jian cheng Bioengineering Institute (Nanjing, China). Using spectrophotometry (BIO-RAD 680), the enzymatic activities were analysed. Using bovine serum albumin as a reference, Bradford’s Coomassie Brilliant Blue (G-250) was used to calculate the protein content. Units of activity per milligram of protein (U/mg protein) were used to express enzyme activities. The second portion was centrifuged at 700 g to separate the plasma and haemocytes, which were then stored at -80°C to be measured later for average protein content, AKP activation, ACP, T-AOC, T-SOD, LZM, NOS, GSH-Px and CAT activation.

### 2.9 Transcriptome sequencing and analysis

The total RNA was extracted using the Trizol reagent in accordance with the usual protocol. Before producing the libraries for Illumina sequencing, the RNA concentration and purity were measured using a NanoDrop ND1000 spectrophotometer (NanoDrop Technologies Inc., DE, USA), the RNA quality was tested using 1% agarose gels, and the RNA integrity was tested on an Agilent 2100 Bioanalyzer (Agilent Technologies, CA, United States). Poly (A) + mRNA selection was performed on one RNA pool using oligo (dT) magnetic beads (Invitrogen, United States). The RNA sequencing libraries were created using an Illumina Tru-Seq™ RNA Sample Preparation Kit (Illumina, San Diego, CA, USA) according to the manufacturer’s instructions. The cDNA libraries obtained were directly used for cluster generation and sequencing on an Illumina HiSeqTM2500 Analyzer at Biomarker Technologies Co., Ltd (Peking, China). NGSQC Toolkits were utilised to filter reads with primer or joint sequence contamination or low quality for quality control of original sequencing reads. The raw reads were first filtered to remove adaptors, ambiguous reads (those with ≥10% ambiguous nucleotides), and low-quality reads (those with ≥20% bases with quality scores of ≤10) before being assembled using Trinity, with min_kmer_cov set to 2 and other parameters set to defaults. Using KAAS, the resulting contigs and unigenes were aligned to the non-redundant (NR) protein database, Gene Ontology (GO), and Kyoto Encyclopedia of Genes and Genomes (KEGG), with an E-value cut-off of 10^−5^.

#### 2.9.1 Library preparation and RNA sequencing

In each example, three biological replicates of transcriptome sequencing were obtained, and all raw data was deposited in the NCBI sequence. Using oligo (dT) magnetic beads, mRNA was isolated from total RNA and broken into small pieces using fragmentation buffer. After cDNA purification with the QIAquick PCR Purification Kit (Qiagen, Hilden, Germany), a cDNA library compatible with Illumina NGS technology was created from the fragmented mRNA by reverse transcription, second-strand synthesis, and ligation of appropriate adapters (paired-ends). The amount of cDNA in each library was determined using the Qubit device and spectrofluorometric analysis. Illumina HiSeq™ 4000 technology was used to obtain the paired-end 100 bp (PE100) data.

#### 2.9.2 Differentially expression analysis

To compare the expression levels in each sample and identify the differentially expressed genes (DEGs), a differential expression analysis test was utilised. To quantify expression abundance, read counts were normalised as FPKM (Fragments Per Kilobase of exon model per Million mapped reads) values for calculating and comparing gene expression variations between individual samples. The FPKM of genes in haemocytes was then statistically analysed using DEseq 1. To determine DEGs in haemocytes in each group, absolute values of log 2 (fold change, FC) > 1 and FDR (false discovery rate) < 0.05 were set as threshold criteria.

To collect annotation information on DEGs, the Basic Local Alignment Search Tool (BLAST) software was used to sequence the DEGs using NR, Swiss-Prot, GO, Clusters of Orthologous Groups of Genes (COG), and KEGG. DEGs were identified by comparing the data to the *H. discus hannai* transcriptome as well as the GO and KEGG and COG databases using BLAST-based searches (E < 10^-5^). The techniques for GO and KEGG pathway annotation were used according to Shen et al. (31). The following processes were used for GO annotation: target genes were extracted, prepared by sequence alignment, assembled, and submitted to a BLAST of GO terms (mapping). Only the GO terms with the pre-set scores were assigned to the target protein sequences. As a consequence, the results of the GO annotation were acquired. The KEGG pathway annotation procedure was as follows: the protein sequence was matched using the KEGG database to produce homologous KEGG genes, and the genes were screened using a bi-directional hit rate to obtain orthologous candidate genes. Based on the probability and heuristic scoring, the data were analysed by the KEGG Orthology (KO) method in order to generate the KO ranking chart. Fisher’s exact test was used to determine the significance degree of protein enrichment of GO terms and each pathway in this study.

### 2.10 Statistical analysis

For paired sample comparisons, a two-tailed Student’s *t*-test was used in this investigation. The SPSS 15.0 program was used for all statistical analyses, and quantitative data were given as means ± SEM. The significance threshold was chosen at *p* ≤ 0.05.

## 3 Results

### 3.1 Haemocyte morphology

**Figure 1:**
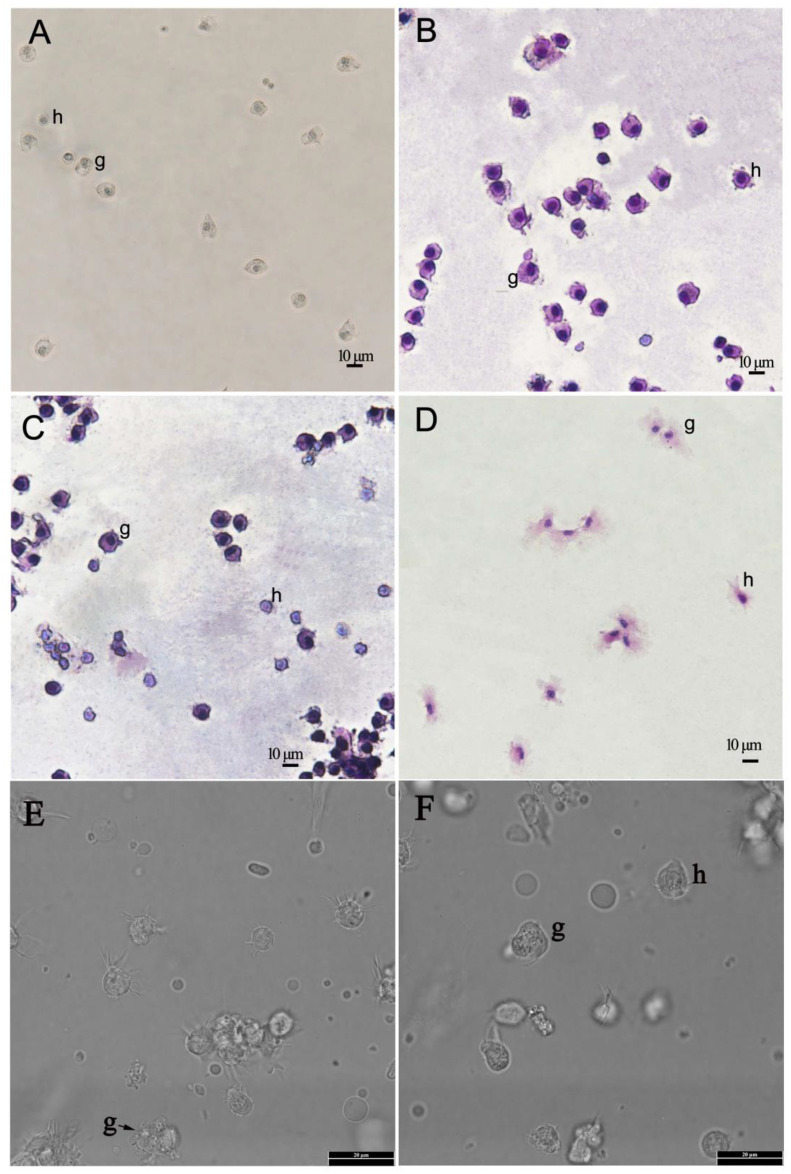
Light micrographs of haemocytes of Pacific abalone (*Haliotis discus hannai*) prepared by spontaneous adhesion using filtered sterilised seawater. **(A)**. The micrographs of unstained haemocytes under the light microscope (200×). **(B, C)**. The micrographs of haemocytes with Wright-Giemsa stain under the light microscope (200×). **(D)**. The micrographs of haemocytes with haematoxylin-eosin staining under the light microscope (200×). **(E, F)**. The micrographs of unstained haemocytes under the light microscope (1000×). h- hyalinocytes, g-granulocytes.

In fixed preparations two kinds of cells were clearly separated by their size and nucleus/cytoplasm (N/C) ratio. The granulocytes had a low N/C ratio. The lowly abundance of small haemocytes with a high N/C ratio are most likely hyalinocytes, as shown in other mollusks (**Figure 1**). Staining of spontaneous adherent or cyto-centrifuged cells with Wright-Giemsa and haematoxylin-eosin revealed two haemocyte types: granulocytes (g) and hyalinocytes (h), depending on the presence or absence of cytoplasmic granules (**Figures 1B–F**). Granulocyte endoplasm had cytoplasmic granules, whereas hyalinocytes had few or none. Granulocytes ranged in size from 8 to 13 μm and had a low N/C ratio. These cells were round or ovoid in shape, with nuclei that were centrally located, round or oval in shape (**Figure 1**).

### 3.2 Non-adherent haemocyte transmission electron microscopy

**Figure 2:**
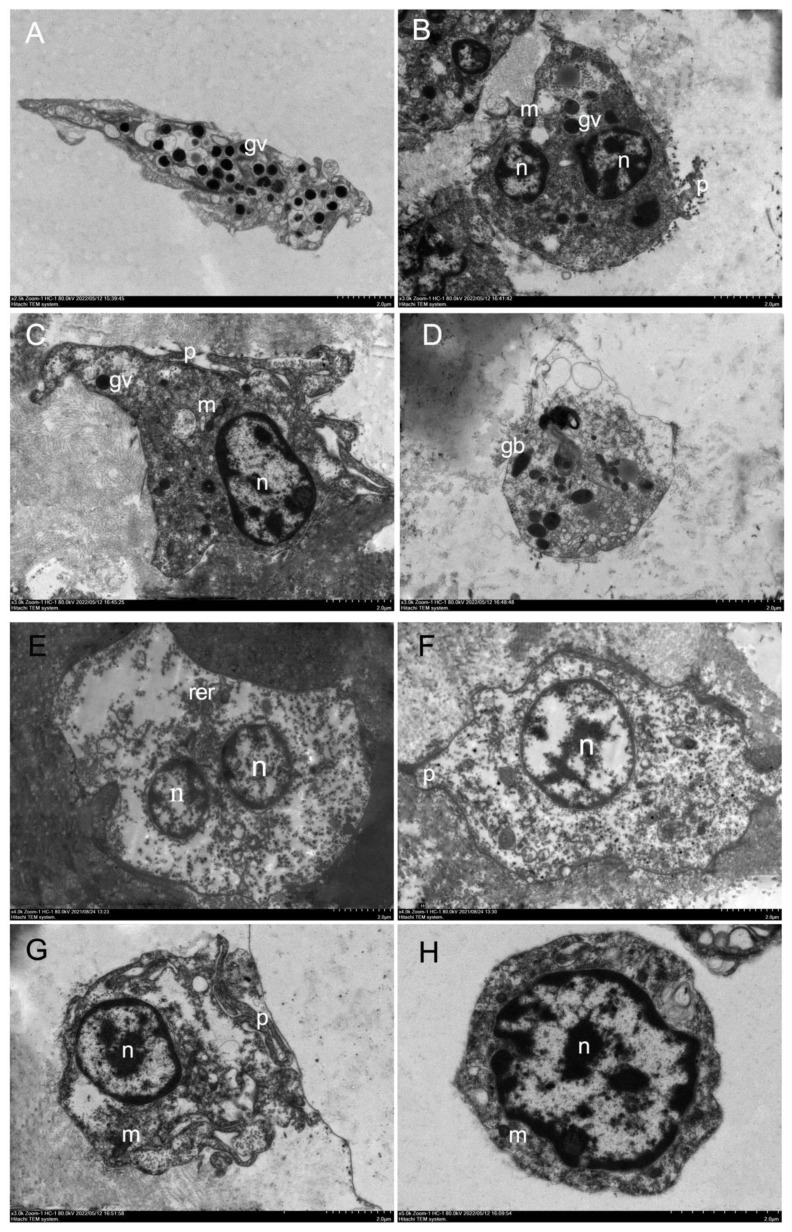
Electronic microscopy of haemocytes in *H*. *discus hannai*. **(A)** Electron microscopy of type I granulocytes in *H. discus hannai*, spherical or oval cells containing many large oval granules, 0.3–0.6 μm in diameter, granulocytes with asymmetrical shape; **(B, C)** electronic microscopy of type II granulocytes in *H*. *discus hannai*; **(D)** type II granulocytes, oval and small nucleus. Rotund or oval granule (gv); nucleus (n); mitochondria (m); pseudopodia (p); bacilliform granule (gb); the letters represent the same meaning in following figure. **(E-H)** Electron microscopy of hyalinocytes in *H. discus hannai*. Electron transmission microscopy of hyalinocytes in *H*. *discus hannai*. Hyalinocytes with asymmetrical shape, pseudopodia can be observed in some hyalinocytes, they have one or several nucleus, and a cytoplasm containing few or no granules, the nucleus was either in a central or an eccentric position; **(E-G)** large hyalinocytes, haemocyte with large nucleus, a small amount of cytoplasm, a small number of and mitochondria in the cytoplasm, hyalinocytes showing pseudopodia; **(H)** cells with a large nucleus, containing a great number of mitochondria, are small hyalinocytes. Nucleus (n); mitochondria (m); pseudopodia (p); rough endoplasmic reticulum (rer).

Comparison of hyalinocytes and granulocytes, granulocytes contained an abundance of electron-dense cytoplasmic particles surrounded by membranes, known as cytoplasmic granules, with sizes ranging from 0.2 to 1.0 μm. The cytoplasm included a variable number of mitochondria, the Golgi complex, endoplasmic reticulum, and small electron-lucid vesicles of various sizes, some of which were most likely derived from the Golgi complex or the smooth endoplasmic reticulum. In this study, granulocytes were classified into two categories based on the number of granules and the morphology of granules: type I granulocytes (**Figure 2A**) and type II granulocytes (**Figures 2B–D**). In the cytoplasm of type I granulocytes, there were many granules, each around 0.5 μm in diameter and oval. Type II granulocytes contained a few granules, of various shapes. Granulocytes however composed the minority (less than 30% on cytocentrifuged cells were observed).

In this study, the hyalinocytes lacked or had few cytoplasmic granules, and the nucleus was either central or eccentric (**Figures 2E–H**). The cytoplasm was filled with a variety of mitochondria and tiny electron-lucid vesicles of varying sizes (**Figure 2G**). The hyalinocytes exhibited well-developed pseudopodia (**Figures 2F, G**); the shape of the hyalinocytes revealed a spreading pseudopodia morphology. There are two types of hyalinocytes based on their size: large (7–10 μm) and small (4–6 μm) hyalinocytes. The large cells had big cytoplasmic vacuoles that did not stain, as seen in live adherent haemocytes (**Figure 2G**). Small hyalinocytes were haemocytes with a big nucleus and a small amount of cytoplasm containing a large number of mitochondria (**Figure 2H**), this resulted in a relatively high N/C ratio. They relate to what are known as blast-like cells (**Figure 2H**). Hyalinocytes composed major part of the haemocyte population (more than 60% on cytocentrifuged cells).

### 3.3 Assessment of haemocytes phagocytosis using electron microscopy

**Figure 3:**
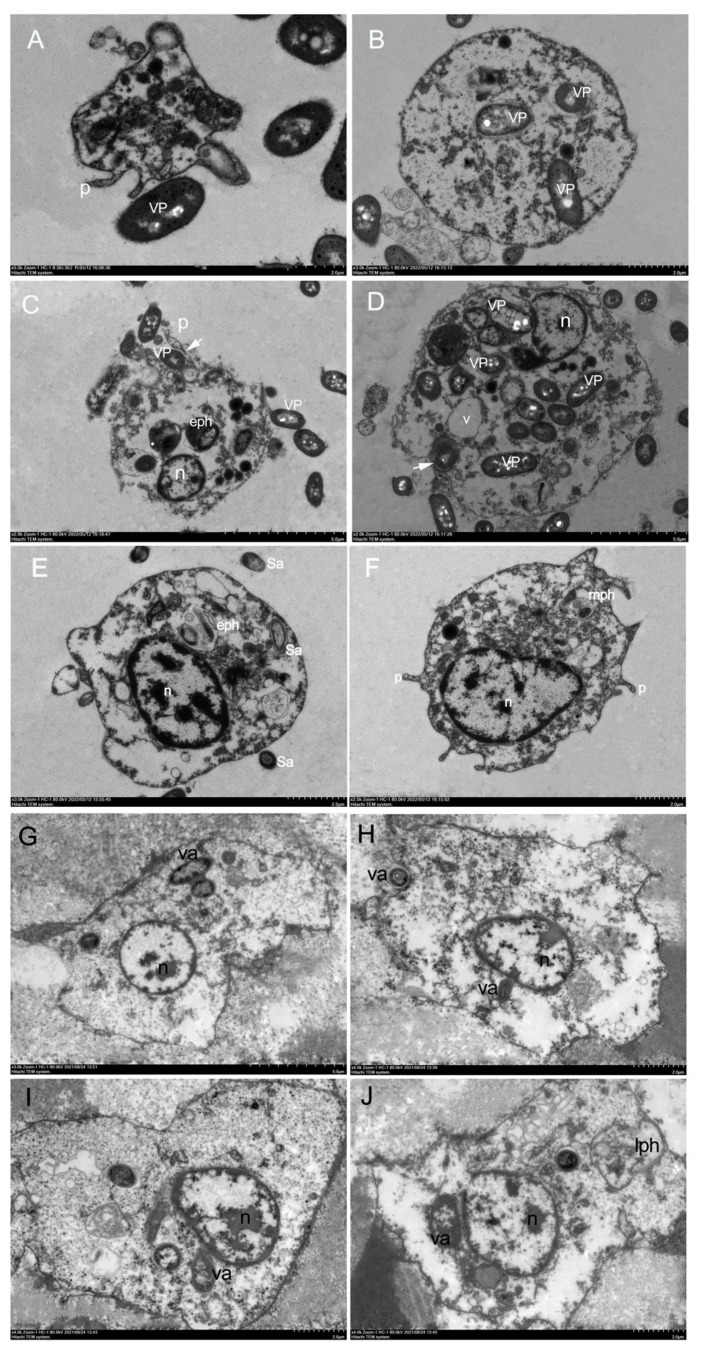
*In vitro* phagocytosis by *H. discus hannai* haemocytes under transmission electron microscopy. **(A-D)**.*In vitro* phagocytosis of *Vibrio parahaemolyticus* cells by *H. discus hannai* haemocytes. **(A)**. Hyalinocytes with pseudopodia, hyalinocytes adhering to bacteria. **(B)**. Hyalinocytes with phagocytising the bacterium. **(C)**. Hyalinocytes with early phagosome, early phagosome containing morphologically intact bacteria. **(D)**. adhering to bacteria and phagocytising the bacterium. n, nucleus; VP, *V. parahaemolyticus*; p, pseudopodia; eph, early phagosome. **(E, F)**. *In vitro* phagocytosis of *S. aureus* cells by *H. discus hannai* haemocytes under transmission electron microscopy. **(E)**. Hyalinocytes with early phagosome, early phagosome containing morphologically intact bacteria. **(F)**. Hyalinocytes with pseudopodia and with the stage between early phagosome and late phagosome (mph). n, nucleus; Sa, *S. aureus*; **(G-J)**. *In vitro* phagocytosis of *Vibrio alginolyticus* cells by *H. discus hannai* haemocytes under transmission electron microscopy. **(G)**. Hyalinocytes with phagocytising the bacterium. **(H, I)**. Hyalinocytes adhering to bacteria and phagocytising. **(J)**. Hyalinocytes with late phagosome. n, nucleus; va, *Vibrio alginolyticus*; lph, late phagosome.

The phagocytosis of haemocytes was studied at a ratio of 1:10 of haemocytes to *V. parahaemolyticus* or *V. alginolyticus* or *S. aureus*. The results of haemocytes that engulfed and internalised *V. parahaemolyticus* or *S. aureus* or *V. alginolyticus* are shown in **Figure 3**, respectively. *In vitro* investigation of phagocytosis of bacteria by *H. discus hannai* haemocytes revealed that haemocytes could phagocytise *V. parahaemolyticus* or *V. alginolyticus* or *S. aureus* under transmission electron microscopy (**Figure 3**). The morphological characteristics of *H. discus hannai* haemocytes differed significantly. The majority of the bacteria were attached to the surface of haemocytes. Phagocytising to bacteria was also detected in haemocytes (**Figure 3**). Granulocytes were seen adhering to the microorganisms *V. parahaemolyticus* (**Figure 3C**). The presence of multiple long and thin cytoplasmic protrusions or filopodia (**Figures 3A, C**) distinguished these haemocytes. Bacterial trapping by haemocyte pseudopods was detected (**Figures 3C, D**). The early phagosome area was filled with a microgranular substance with a low electron density (**Figure 3C**). Individual phagosomes were used to internalise bacteria (**Figure 3C**).

When haemocytes phagocytised *S. aureus*, the nucleus had an active character, and the heterochromatin was mostly organised in electron-dense clumps and near the nucleus’s periphery (**Figures 3E, F**). During phagocytosis, bacteria stimulated hyalinocytes with phagosomes, including early phagosomes containing morphologically intact bacteria (**Figure 3E**), hyalinocytes with pseudopodia and with the stage between early phagosome and late phagosome (**Figure 3F**).

When haemocytes phagocytised *V. alginolyticus*, the haemocytes changed shape and the appearance of pseudopods was seen, as well as endocytosis of the bacterium *V. alginolyticus* (**Figures 3G–J**). Only a small percentage of granulocytes were capable of phagocytising *V. alginolyticus*. One or more whole bacteria might be phagocytised by the haemocytes. Bacteria became attached to the surface of the haemocytes, resulting in an attachment phenomenon (**Figure 3H**), late phagosomes with only debris, possibly bacterial fragments (**Figure 3J**). The findings revealed that the phagocytosis capacities of *H. discus hannai* hyalinocytes and granulocytes to *V. alginolyticus* could potentially be different. This suggested that phagocytic haemocytes are most likely hyalinocytes, but granulocytes can also be phagocytic, however electron microscopy may not detect them.

### 3.4 Phagocytosis and THC by flow cytometry in haemocytes infected by bacteria

**Figure 4:**
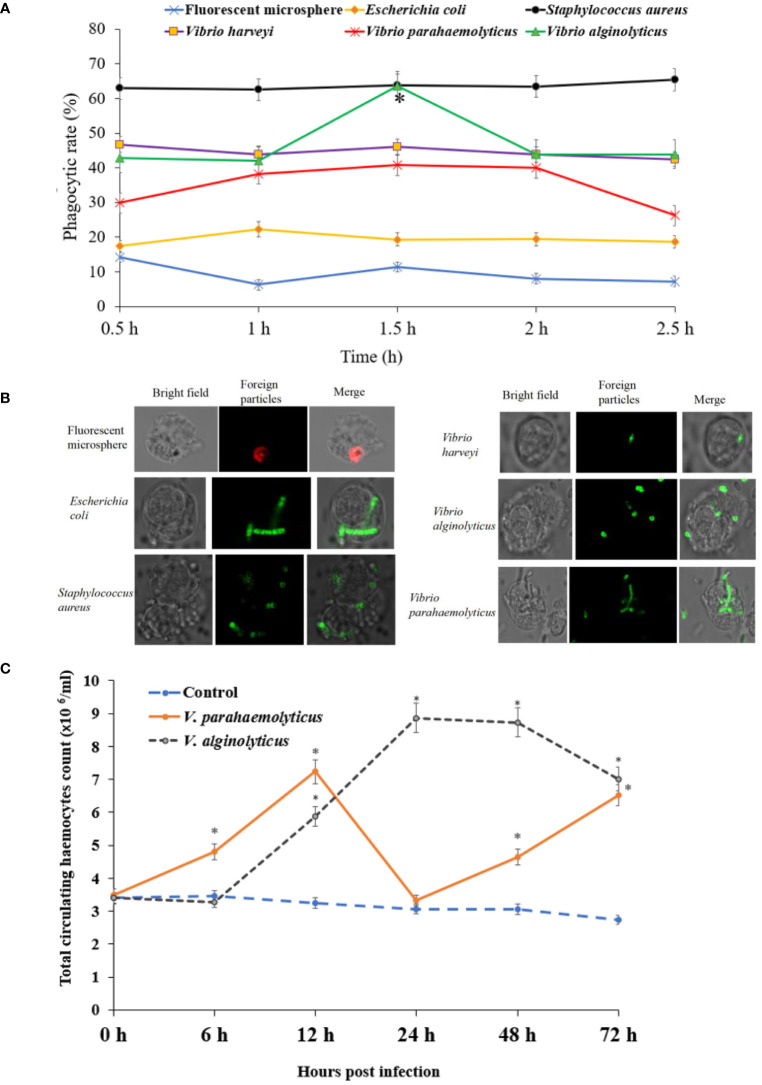
Phagocytic capability of haemocyte detected by flow cytometry in *H. discus hannai.* **(A)** The phagocytosis of haemocytes was detected by flow cytometry (bacteria were labelled with FITC). **(B)** The fluorescence microscope images of phagocytosis. **(C)** Total circulating haemocytes count of haemocytes to *Vibrio alginolyticus* or *Vibrio parahaemolyticus* by foot injection.

*In vitro* phagocytosis experiment, flow cytometry was utilised to detect haemocyte phagocytosis of fluorescent beads. The fluorescent beads’ phagocytic rates in haemocytes were measured (**Figures 4A, B**). The phagocytic rate (sometimes known as the phagocytic percentage, PP) was higher at 0.5 h, which was 14.35%. The greatest increase in PP of *E. coli* occurred at 1 h which was 22.27%, and the PP of *E. coli* in haemocyte increased no further and started to decrease (**Figure 4A**). The PP of *S. aureus* was higher than the other five foreign particles, which was about 63%. The PP of *V. harveyi* was about 43%, it was no significant difference at different time points. The greatest increase in PP of *V. parahaemolyticus* occurred at 1.5 h which was 40.8%, and decreased at 2.5 h. The PP peak of *V. alginolyticus* in haemocyte was 63.7% at 1.5 h.

#### 3.4.1 THC

No abalone was dead during the sub-lethal bacterial *V. parahaemolyticus* or *V. alginolyticus* stress. The THC variation patterns were shown in **Figure 4C**. THC of *V. parahaemolyticus* exposure increased steadily and peaked was 7.23 ×10^6^ cells/mL at 12 h, then decreased significantly at 24 h, subsequently increased steadily at 48 h and 72 h. THC of *V. alginolyticus* exposure increased and peaked was 8.87 ×10^6^ cells/mL at 24 h, then decreased at 48 h and 72 h. THC of control group was relatively stable during the whole period.

### 3.5 Unigene basic annotation using transcriptome analysis after *V. parahaemolyticus* or *V. alginolyticus* stress

#### 3.5.1 Summary of annotation results

**Figure 5:**
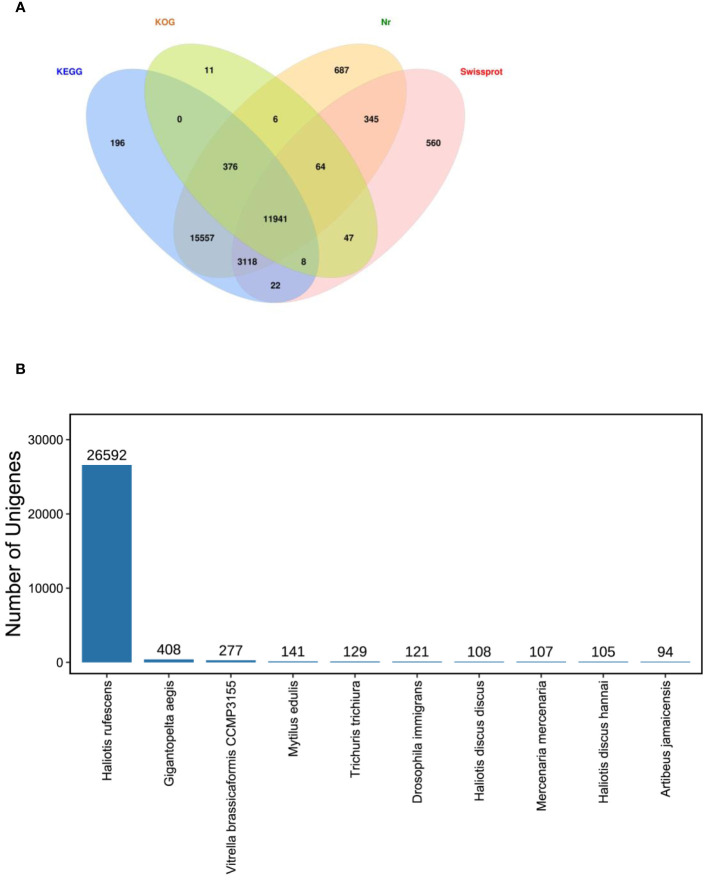
Unigenes annotated. **(A)**. Venn diagram of all unigenes annotated against Nr, SwissProt, KOG and KEGG databases in the Pacific abalone (*Haliotis discus hannai*) in response to *V. parahaemolyticus* and *V. alginolyticus* stress. The number in each colour block indicated the number of unigenes that was annotated by single or multiple databases. **(B)** Species distribution of sequences matched to the Nr database.

Total 86235 unigenes were annotated by Nr, KOG, KEGG and Swiss-Prot database. Of them, 32094,12453, 31218, 16105 unigenes were annotated by Nr, KOG, KEGG and Swiss-Prot database, respectively. Annotation genes were 32938 (**Figure 5A**).

#### 3.5.2 Functional annotation of non-redundant unigenes

In this study, the majority annotated unigenes matched *Haliotis rufescens* (**Figure 5B**). In the species distribution of sequences matched to the Nr database, the unigenes revealed similarities to sequences of *H. rufescens* (26592 unigenes), *Gigantopelta aegis* (408), *Vitrella brassicaformis CCMP3155* (277), *Mytilus edulis* (141), *Trichuris trichiura* (129), *Drosophila immigrans* (121), *Haliotis discus discus* (108), *Mercenaria mercenaria* (107), *H. discus hannai* (105).

#### 3.5.3 KEGG annotation

**Figure 6:**
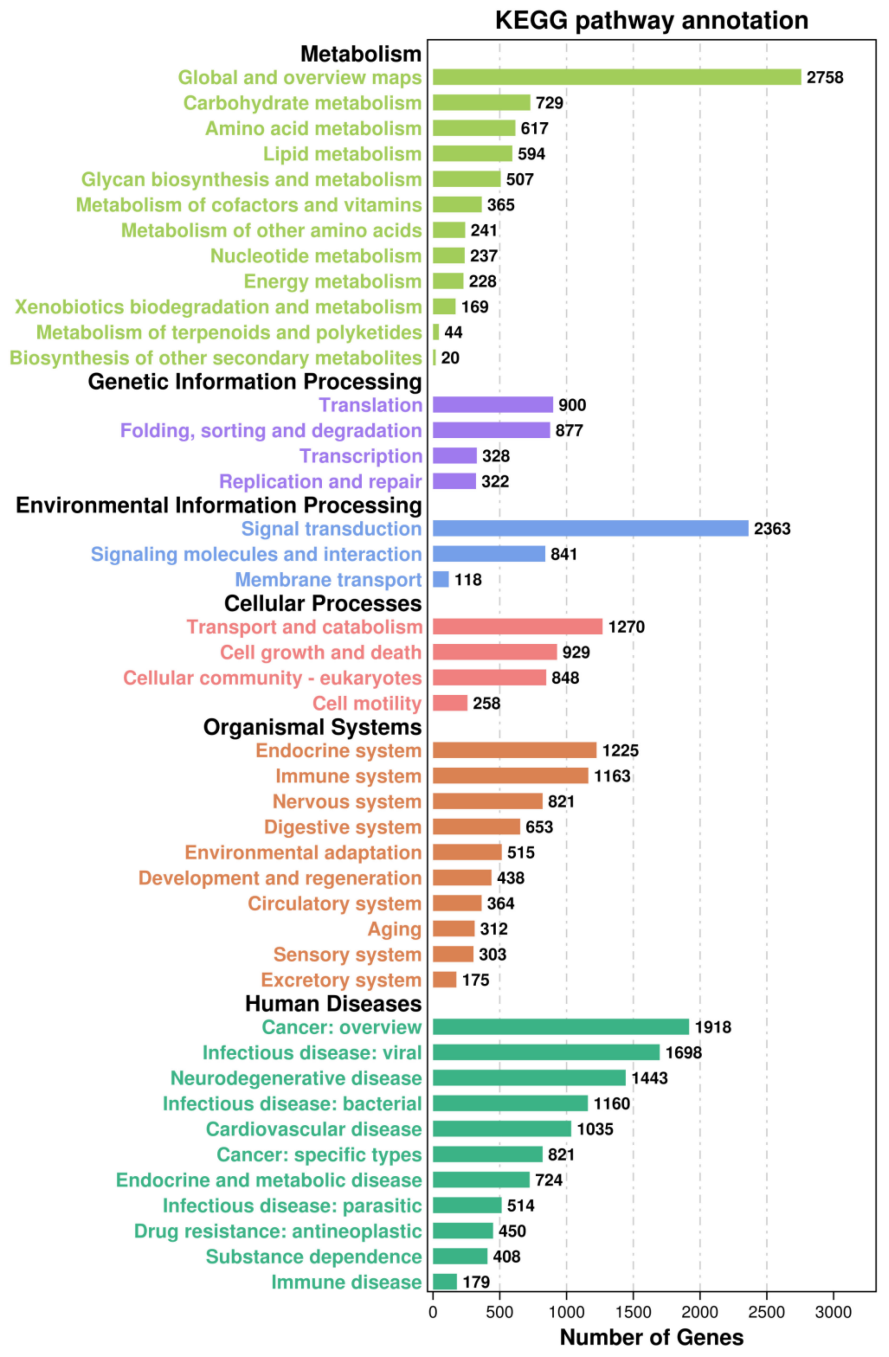
The Kyoto Encyclopedia of Genes and Genomes (KEGG) classification of all assembled unigenes in the Pacific abalone (*H. discus hannai*).

What we were interested in is the immune-related pathways, as shown in **Figure 6**, the majority of immune-related genes were classified into pathways for cell growth and death (929 genes) [including apoptosis (340 genes), necroptosis (237)], cellular community - eukaryotes (848) [including focal adhesion (393)], immune system (1163) [including NOD-like receptor signalling pathway (219), C-type lectin receptor signalling pathway (167), Toll and Imd signalling pathway (145), chemokine signalling pathway (141), Toll-like receptor signalling pathway (139)], signal transduction (2363) [including PI3K-Akt signalling pathway (452), MAPK signalling pathway (317), Ras signalling pathway (303), Rap1 signalling pathway (301), mTOR signalling pathway (230), HIF-1 signalling pathway (185), Wnt signalling pathway (181), TNF signalling pathway (178)], signalling molecules and interaction (841)[including cell adhesion molecules (195)], transport and catabolism (1270) [including lysosome (318), endocytosis (308), autophagy - animal (252), phagosome (244)].

#### 3.5.4 COG/KOG

**Figure 7:**
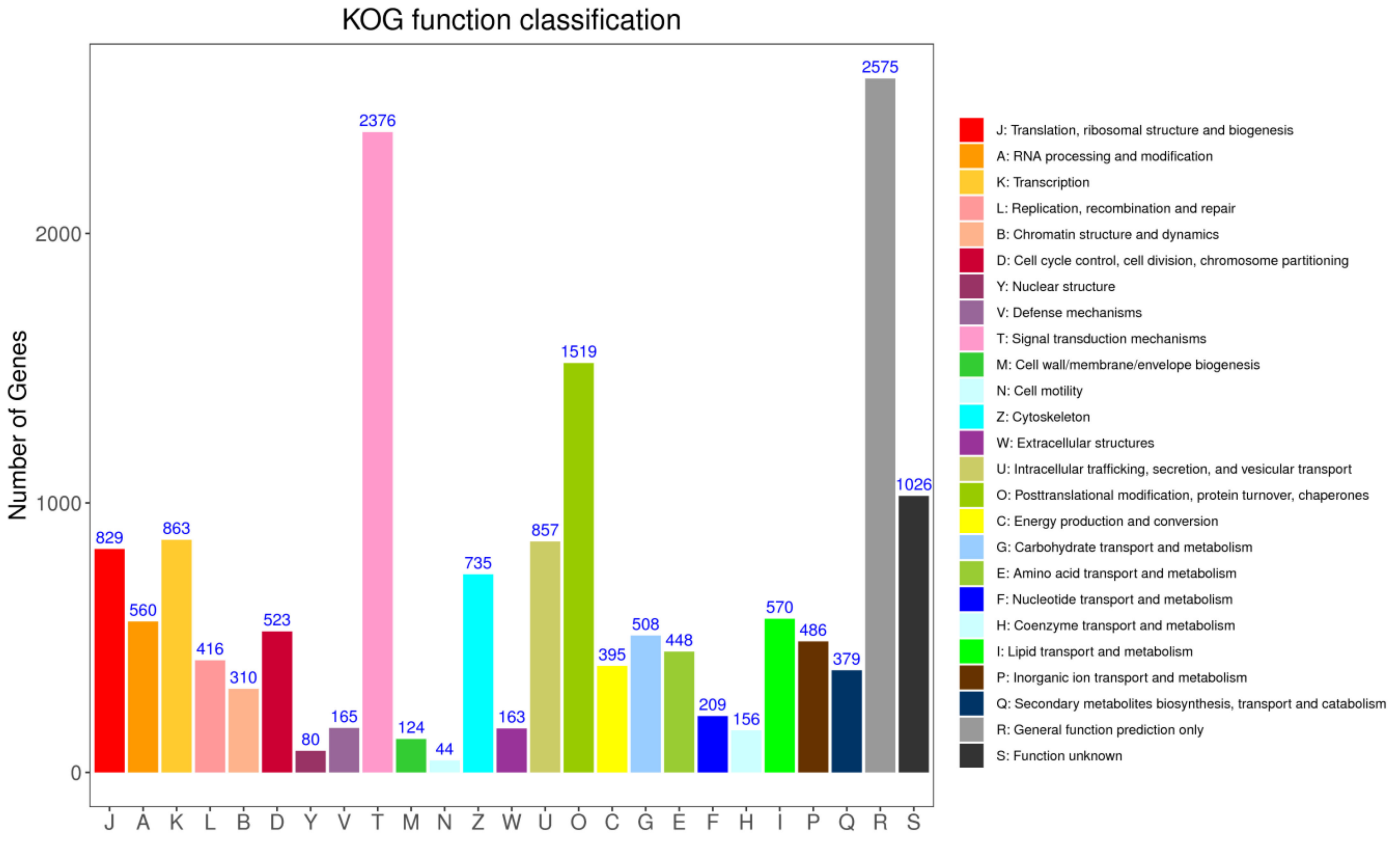
The cluster of orthologous groups (COG) classification. A total of 16316 unigenes were annotated into 25 categories.

KOG annotation identified 16316 putative genes across 25 categories (**Figure 7**). Among these categories, the cluster for “signal transduction mechanisms (2376 genes)” represented the second largest group, followed by posttranslational modification, protein turnover, chaperones (1519), in addition, the number of proteins involved in defence mechanisms was 165 genes.

#### 3.5.5 GO annotation

**Figure 8:**
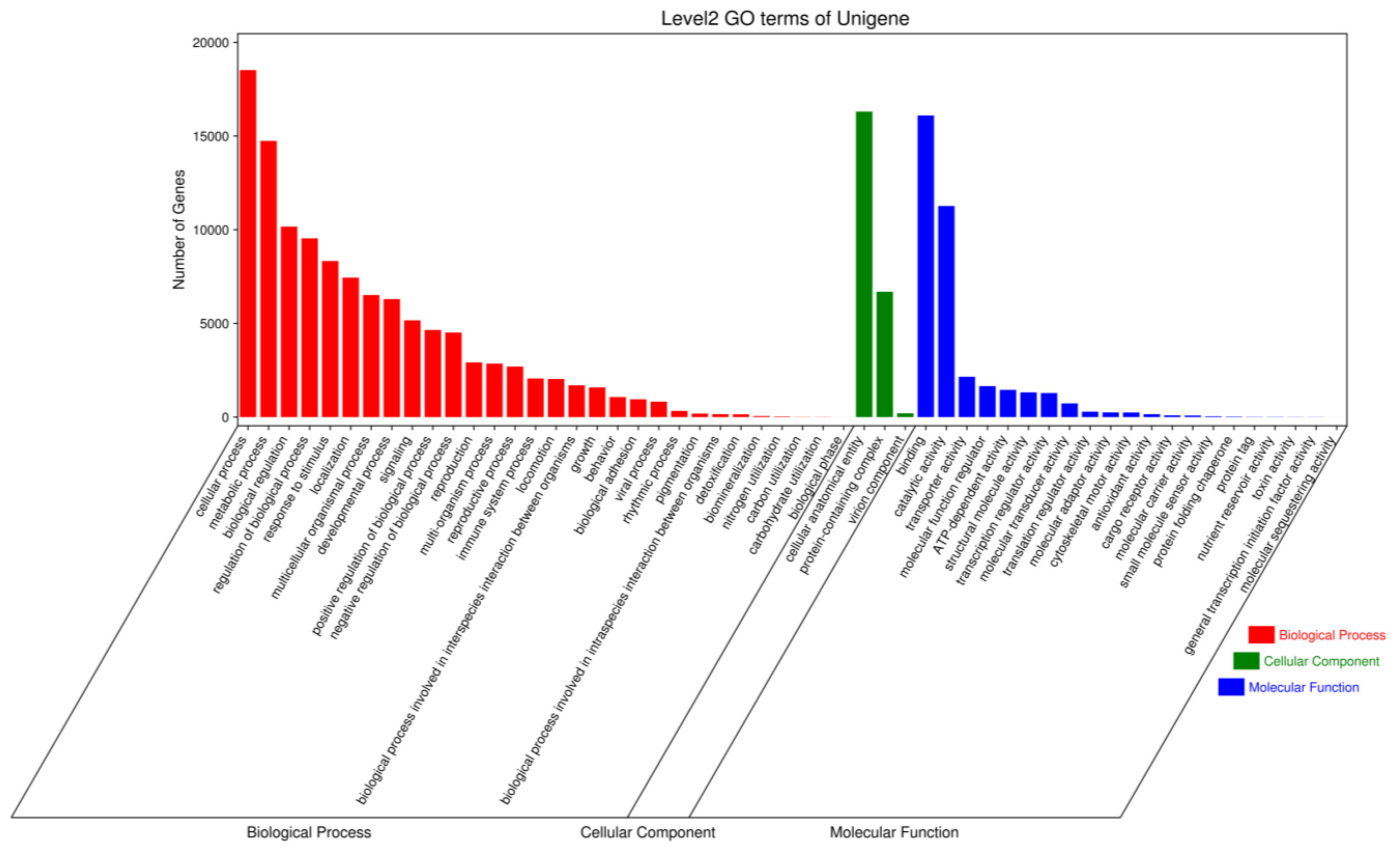
GO function classification of the Pacific abalone (*H. discus hannai*) in response to *V. parahaemolyticus* and *V. alginolyticus* stress.

GO classifies genes and gene products into three categories: biological processes (BPs), cellular components (CCs) and molecular functions (MFs). In three samples, the discovered proteins were examined using GO annotations; the GO functional categorisation is presented in **Figure 8**. Using the biological process, the genes participating in cellular process (18519 unigenes), metabolic process (14742 unigenes), biological regulation (10164), regulation of biological process (9540), response to stimulus (8329), immune system process (2060), viral process (823). Using the molecular function term, the genes involved in binding (16098), catalytic activity (11266), transporter activity (2152), molecular function regulator (1652), ATP-dependent activity (1453), antioxidant activity (154). Using the cellular component term, the number of proteins involved in cellular anatomical entity (16305), the first largest, followed by those related to protein-containing complex (6692), virion component ((203), respectively.

### 3.6 Transcriptomic differences in haemocytes challenged by *V. parahaemolyticus* and *V. alginolyticus*

Differential expression of unigenes found in five groups was assessed by computing FPKM values obtained by matching the high quality reads to a reference transcriptome constructed by clustering three of the samples unigenes. Transcripts were classed as up or down regulated based on their log fold change (FC) value, which was determined using the FC= Log2 (Treated/Control) algorithm. FC values larger than 1 were regarded to be up-regulated, whereas values less than 1 were thought to be down-regulated. We utilised *p*-value 0.05 and |log2 (fold change)|> 1 to look for up or down regulated genes.

**Figure 9:**
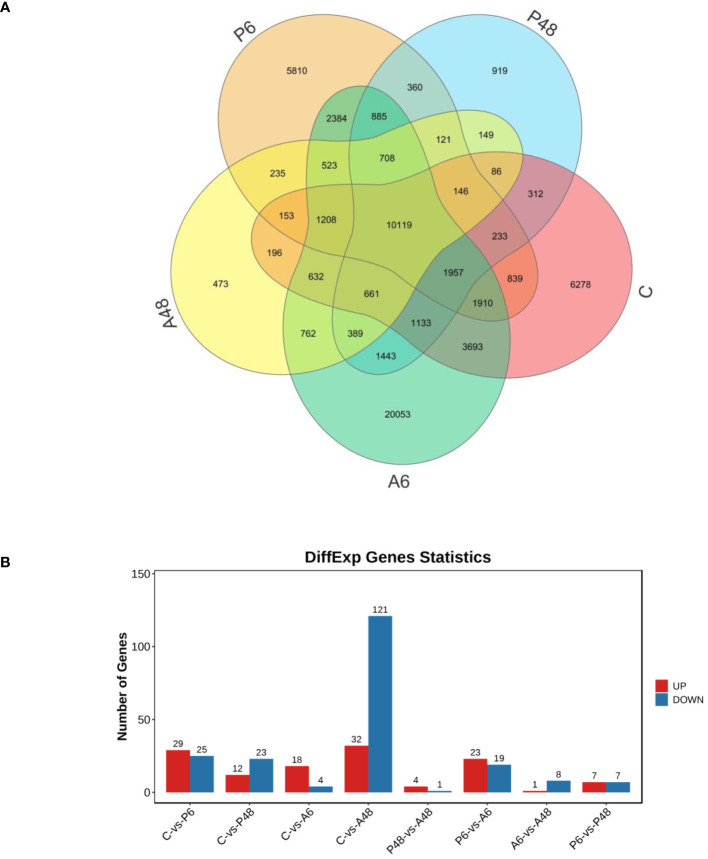
Venn diagram of all genes and statistics of different expression genes. **(A)**. Venn diagram of all genes. **(B)** Statistics of different expression genes. Genes in control group **(C)**, haemocytes challenged by *V. parahaemolyticus* for 6 h (P6), haemocytes challenged by *V. parahaemolyticus* for 48 h (P48), haemocytes challenged by *V. alginolyticus* for 6 h (A6), and haemocytes challenged by *V. alginolyticus* for 48 h (A48), respectively.

**Figure 9** showed gene information discovered using transcriptomic approaches. Using an organism database, bacterial genes were removed from the analysis. After a 6-hour and 48-hour *V. parahaemolyticus* stress, 29 and 12 genes were significantly up-regulated in P6 and P48, respectively; 25 and 23 genes were significantly down-regulated in P6 and P48, respectively. After *V. alginolyticus* challenge for 6 h and 48 h, 18 and 32 genes were significantly up-regulated in A6 and A48, respectively, 4 and 121 genes were significantly down-regulated in A6 and A48, respectively.

In comparing differentially expressed genes among groups, we focused on immune-related genes. After *V. alginolyticus* challenge 6 h, in comparisons of C and A6, it was worth noting that deleted in interleukin-6 receptor subunit beta-like and protein turtle homolog B-like were present in significantly higher levels in A6 group (**Supplementary Table 1**). After *V. alginolyticus* challenge 48h, in comparisons of C and A48, most of the differentially expressed genes were significantly down-regulated in A48, especially rho GTPase-activating protein 6-like isoform X2, leukocyte surface antigen CD53-like, calponin-1-like, calmodulin-like, troponin C, troponin I-like isoform X4, troponin T-like isoform X18, tumor necrosis factor ligand superfamily member 10-like (**Supplementary Table 1**). However, rho-related protein racA-like and haemagglutinin/amebocyte aggregation factor-like were significantly up-regulated in A48 (**Supplementary Table 1**).

After *V. parahaemolyticus* challenge 6h, compared the C and P6 groups, haemagglutinin/amebocyte aggregation factor-like was significantly up-regulated in P6 (**Supplementary Table 1**). However, supervillin-like isoform X4 was expressed at significantly lower levels in P6 (**Supplementary Table1**). However, after *V. parahaemolyticus* challenge 48 h, calmodulin-like and kyphoscoliosis peptidase-like were considerably lower in the P48 group (*p*< 0.05) (**Supplementary Table 1**). The observed differentially expressed genes in haemocytes may have a tight relationship with their function features.

### 3.7 Gene Ontology enrichment analysis of differentially expressed genes

**Figure 10:**
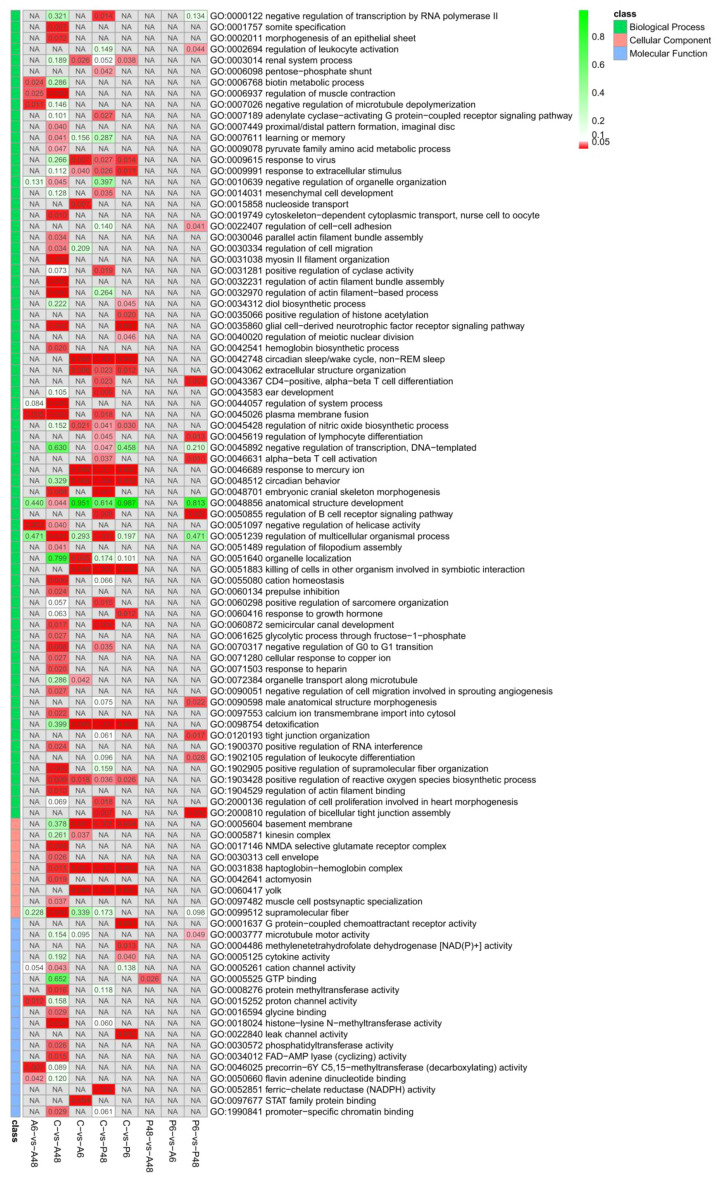
Significantly enriched GO taxonomy of differentially expressed genes (DEGs) in the comparisons of five groups (*p* value<0.05).

GO enrichment analysis for the DEGs was performed to characterise the expression changes in the five samples with the whole transcriptome dataset as the background to gain insight into the functional categories that were altered between healthy and *V. parahaemolyticus*-infected haemocytes and between healthy and *V. alginolyticus*-infected haemocytes. GO annotations were used to classify the discovered genes in C and A6, C and A48, C and P6, C and P48. **Figure 10** showed the GO functional categorisation analysis.

Based on biological processes, the DEGs were assigned to GO categories. In the comparison of the C and A6 groups, most of the up-regulated genes were involved in binding (including 7 genes), cellular anatomical entity (7 genes), cellular process (6 genes), catalytic activity (5 genes), metabolic process (5 genes), localisation (5 genes), response to stimulus [3 genes, including Unigene0003336 (*prtgb*:interleukin-6 receptor subunit beta-like)], antioxidant activity (2 genes), detoxification(2 genes), immune system process (1 genes, which was *prtgb*) in the A6 group. The numbers of largely down-regulated genes participating in these biological process categories were 2, 1, 1, 0, 1,1,1,0 and 1, respectively in the A6 group (**Figure 10**; **Supplementary Table 2**).

For the C vs A48 groups, most up-regulated genes were involved in binding (including 10 genes), catalytic activity (7 genes), cellular anatomical entity (6 genes), cellular process (6 genes), response to stimulus [4 genes, including Unigene0002677 (*racA*: rho-related protein racA-like) Unigene0071836 (*dvr1*: protein DVR-1-like) and Unigene0100902(*birc7-a*: baculoviral IAP repeat-containing protein 3-like), immune system process (1 genes, which was *birc7-a*) in the A48 group. The numbers of down-regulated proteins involved in binding (65 genes), cellular anatomical entity (58 genes), cellular process (53 genes), response to stimulus [28 genes, including Unigene0001551 (*tkfc*: triokinase/FMN cyclase-like isoform X1), Unigene0029628 (*fhod3*: FH1/FH2 domain-containing protein 3-like), Unigene0030552 (*para*: myosin heavy chain, striated muscle-like isoform X10), Unigene0031634 (*ryr*: ryanodine receptor 2-like isoform X17), Unigene0035336 (*magi1*: membrane-associated guanylate kinase), Unigene0037904 (*tni-4*: troponin I-like isoform X4), Unigene0042150 (*arhgap6*: rho GTPase-activating protein 6-like isoform X2), Unigene0060888 (*sqh*: myosin regulatory light chain 12A-like),Unigene0061241(*tspan4*: leukocyte surface antigen CD53-like), Unigene0069048 (*tbx1-b*: T-box transcription factor TBX1-like), Unigene0069977 (*nox5*: NADPH oxidase 5-like isoform X2), Unigene0077316 (*cd40lg*: tumor necrosis factor ligand superfamily member 10-like), Unigene0081343 (*aoc1*: putative amine oxidase), Unigene0081566 (*mid1*: E3 ubiquitin-protein ligase Midline-1-like isoform X3), Unigene0096861(*sfrp1*: secreted frizzled-related protein 1-like isoform X1), immune system process (12 genes), biological adhesion (5 genes) in the A48 group (**Figure 10**; **Supplementary Table 2**).

For the C vs P6 groups, the up-regulated genes were involved in binding (5 genes), cellular anatomical entity (5 genes), metabolic process (4 genes), cellular process (4 genes), response to stimulus (3 genes, including *dvr1*), antioxidant activity (2 genes), detoxification (2 genes) in the P6 group. The numbers of down-regulated genes involved in these biological process categories were 8, 8, 5, 7, 4, 0 and 1, respectively, in the P6 group (**Figure 10**; **Supplementary Table 2**).

For the C vs P48 groups, up-regulated genes were involved in metabolic process (4 genes), catalytic activity (3 genes), binding (3 genes), response to stimulus (3 genes), antioxidant activity (2 genes) in the P48 group. The numbers of down-regulated genes involved in the binding (13 genes), cellular anatomical entity (13 genes), cellular process (11 genes), response to stimulus [7 genes, including Unigene0044790 (*steap4*, metalloreductase STEAP4-like), *tbx1-b*], immune system process (3 genes, including *tbx1-b*) in the P48 group (**Figure 10**; **Supplementary Table 2**).

### 3.8 KEGG pathway enrichment with differentially expressed genes among samples

**Figure 11:**
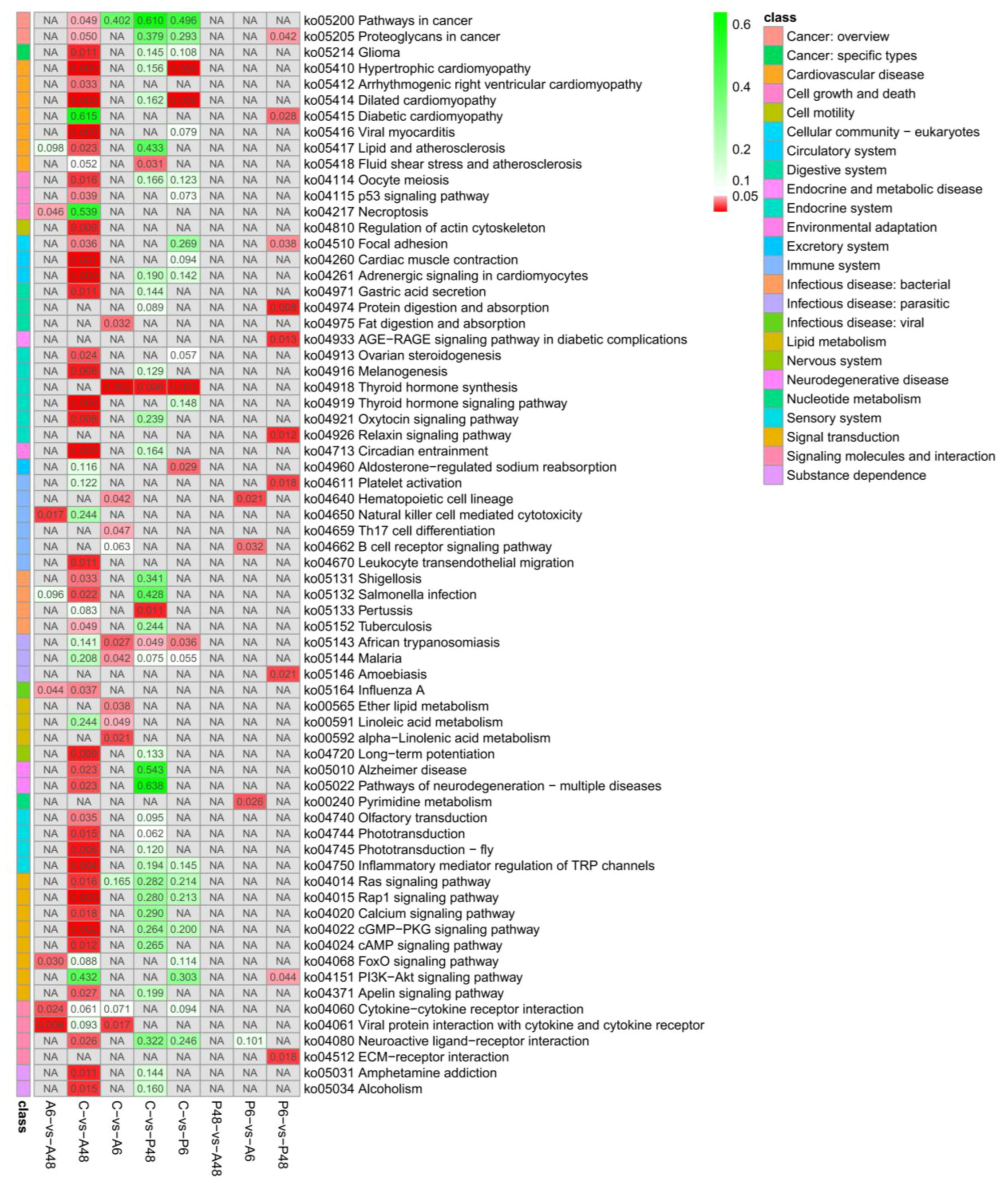
Significantly enriched KEGG pathways of differentially expressed genes (DEGs) in the comparisons of five groups (*p* value<0.05).

For the five samples, significant difference KEGG pathways are shown in **Figure 11**. After *V. alginolyticus* challenge, in comparing C and A6 group, the ko04918 thyroid hormone synthesis, ko04640 haematopoietic cell lineage and ko04659 Th17 cell differentiation signalling pathway had significant difference in A6 (*p* < 0.05). In comparing C and A48 group, ko04810 regulation of actin cytoskeleton, ko04115 p53 signalling pathway, ko04510 focal adhesion, ko04913 ovarian steroidogenesis, ko04916 melanogenesis, ko04921 oxytocin signalling pathway, ko04919 thyroid hormone signalling pathway, ko04713 circadian entrainment, ko04750 inflammatory mediator regulation of TRP channels, ko04670 leukocyte transendothelial migration, ko04015 rap1 signalling pathway, ko04014 ras signalling pathway, ko04020 calcium signalling pathway, ko04024 cAMP signalling pathway, ko04022 cGMP-PKG signalling pathway, ko04080 neuroactive ligand-receptor interaction had significant difference in A48 (p < 0.05). The results showed that after 48 hours of stress with *V. alginolyticus*, there were significant changes in the signalling pathway compared to the control group.

After *V. parahaemolyticus* challenge, in comparing C and P6, ko04918 thyroid hormone synthesis signalling pathway had significant difference in P6. In comparisons of C and P48, ko04918 thyroid hormone synthesis signalling pathway had significant difference in P6 (**Figure 11**).

### 3.9 Series protein expression data analysis by the short time-series expression miner (STEM)+

**Figure 12:**
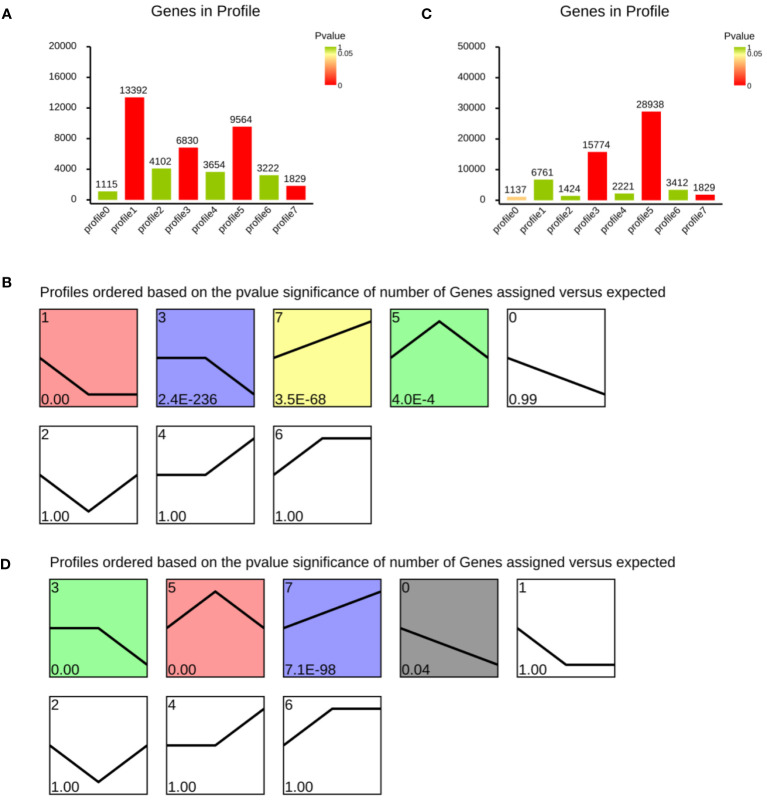
Cluster analysis of differentially expressed genes after *Vibrio parahaemolyticus* and *Vibrio alginolyticus* exposure based on FPKM-means method. **(A, B)** Differentially expressed genes after *Vibrio parahaemolyticus* exposure. **(C, D)** Differentially expressed genes after *Vibrio alginolyticus* exposure. Clusters ordered based on the number of genes. The number in the top left-hand corner refers to profile ID number, and enrichment *p* value appears in the lower-left of each small square. Coloured profiles show statistically significant numbers.

STEM is a program that analyzes short time-series gene expression data. Data from abalone samples (C, A6, A48, P6 and P48) that showed a 1.5-fold increase or decrease in at least one sample were filtered. The findings of FPKM-means clustering were used to partition the DEG expression profiles into eight categories (**Figure 12**). The number in the upper left-hand corner of a profile box reflects the profile ID number, and the enrichment *p* value appears in the bottom left-hand corner of each small square. A statistically significant number of assigned genes is shown in coloured profiles. Non-white profiles of the same colour are clustered together in a single cluster.

**Figure 12A** showed the number of genes in profile after *V. parahaemolyticus* challenge. **Figure 12B** showed a reordered model profile interface. The *p* value was used to arrange the profiles. Profile 1 includes genes that were negatively regulated throughout the *V. parahaemolyticus* challenge phase. Profile 3 contained genes negatively modulated in P48. Profile 5 contained genes were up-regulated in P6. Genes in profile 7 were up-regulated during the whole process (**Figures 12A, B**). Genes in profile 3 and profile 5 were up-regulated after *V. alginolyticus* challenge in A6 group (**Figures 12C, D**). Profile 0 contained genes negatively modulated during the whole process of *V. alginolyticus* challenge. Group 7 contained genes positively modulated.

In C, P6 and P48 group, cathepsin O-like, rab3 GTPase-activating protein, Rab-35-like, SCO-spondin-like, V-type proton ATPase subunit e 2-like, alkaline phosphatase-like were up-regulated after *V. parahaemolyticus* challenge in P6 group; 10 kDa heat shock protein was down-regulated in P48; cathepsin L and cathepsin Z-like were up-regulated in P48; cdc42 homolog was up-regulated during the whole process; cathepsin L-like isoform X1,CCR4-NOT transcription complex subunit 11-like isoform, cold shock domain-containing protein, hemocyanin type 1, mucin-16-like isoform X2, RAB11-binding protein RELCH homolog isoform X2, RAB6-interacting golgin-like, rab9 effector protein with kelch motifs-like, ral GTPase-activating protein subunit beta-like isoform X3, Rab-20-like isoform X1, Rab-3-like, Rab-43-like, Rab-8A-like, Rap1-like, rho GTPase-activating protein 32-like isoform X4 were down-regulated during the whole process (**Supplementary Table 3**).

In C, A6 and A48 group, 1-phosphatidylinositol 3-phosphate 5, activated CDC42 kinase 1, activator of 90 kDa heat shock protein ATPase homolog 1-like, adhesion G protein-coupled receptor, C1q domain containing protein 2, C1q-related factor-like isoform X2, calmodulin-like, cathepsin L1-like, cathepsin B-like, cathepsin O-like, C-type lectin-like isoform X1, Hsp20, heat shock factor protein 1, heat shock 70 kDa protein, heat shock protein 60A-like, histone, interferon, mucin-22-like, mucin-2-like isoform, mucin-3A-like, mucin-4-like isoform X2, mucin-5AC-like, rab9 effector protein, Rab-14, Rab-1A-like, Rab-20-like isoform X1, Rab-3 isoform X1, Rab-32-like isoform X1, Rab-37-like, Rab-39B-like, Rab-40B-like, Rab-43-like, SCO-spondin-like isoform, serine/threonine-protein kinase mTOR-like isoform X2, toll-like receptor were up-regulated after *V. alginolyticus* challenge in A6 group; cathepsin Z-like, cdc42 homolog, heat shock protein HSP 90-beta-like, MAP kinase-interacting serine/threonine-protein kinase 1-like isoform X2, rab5 GDP/GTP exchange factor-like Rab-6B-like, Rab-9A-like were up-regulated in A48;10 kDa heat shock protein, cold shock domain protein, extracellular superoxide dismutase [Cu-Zn]-like isoform X3, ferritin, hemocyanin type 1, NF-kappa-B-activating protein-like, catalase were down-regulated during the whole process (**Supplementary Table 4**).

### 3.10 Predicted interactions of identified DEGs from STEM

**Figure 13:**
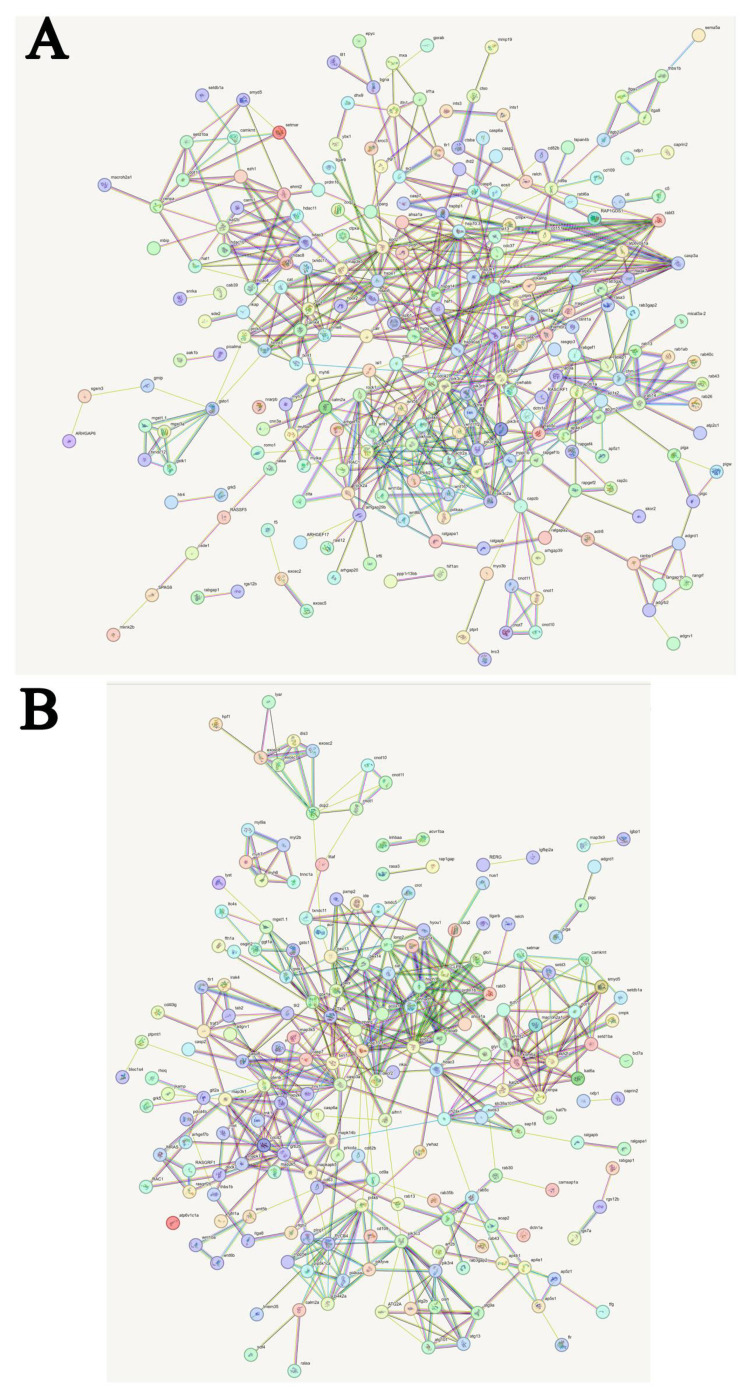
Predicted interactions of identified DEGs. Different line colours represent types of evidence for association. Genes without interactions have been removed from the graph. Genes abbreviations and corresponding full name are shown in **Supplementary Tables 5**, **6**. **(A)** Predicted interactions of identified DEGs in C, A6 and A48 groups; **(B)** Predicted interactions of identified DEGs in C, P6 and P48 groups.

**Figure 13** was obtained from the https://cn.string-db.org/ and shows the predicted interactions of the identified DEGs. In C, A6 and A48 groups, major clusters were associated with microsomal glutathione S-transferase, thioredoxin-like protein, peroxiredoxin-5, catalase, manganese-superoxide dismutase [MnSOD], histone related protein, 10 kDa heat shock protein, heat shock 70 kDa protein, heat shock protein HSP 90, toll-like receptor 13, epidermal growth factor receptor, rab3 GTPase-activating protein, ras-related protein Rab-8A, Rab-14, Rab-37, Rab-43, Rab-9A, RABF1-like, rab5 GDP/GTP exchange factor, ral GTPase-activating protein, cdc42, actin-related protein, serine/threonine-protein kinase mTOR, phosphatidylinositol 4,5-bisphosphate 3-kinase, 1-phosphatidylinositol 4,5-bisphosphate phosphodiesterase, phosphoinositide 3-kinase, protein Wnt-1, protein Wnt-5a, protein Wnt-10a (**Figure 13A**; **Supplementary Table 5**).

In C, P6 and P48 groups, major clusters were associated with exosome complex component, m7GpppN-mRNA hydrolase, CCR4, glutathione-S-transferase, peroxisomal membrane protein, catalase, hypoxia up-regulated protein, stress-70 protein, manganese-superoxide dismutase, Heat shock cognate 70 kDa protein, caspase-3-like, mitogen-activated protein kinase kinase6, cdc42, phosphatidylinositol 4-phosphate 5-kinase, 1-phosphatidylinositol 4,5-bisphosphate phosphodiesterase, phosphatidylinositol polyphosphate 5-phosphatase, rab proteins geranylgeranyl transferase component, ras-related protein RABF1-like, Rab-35-like, Rab-8A-like, Rab-43-like, Rab-30-like, rab3 GTPase-activating protein, histone related protein (**Figure 13B**; **Supplementary Table 6**).

### 3.11 Enzyme activities analysis

**Figure 14:**
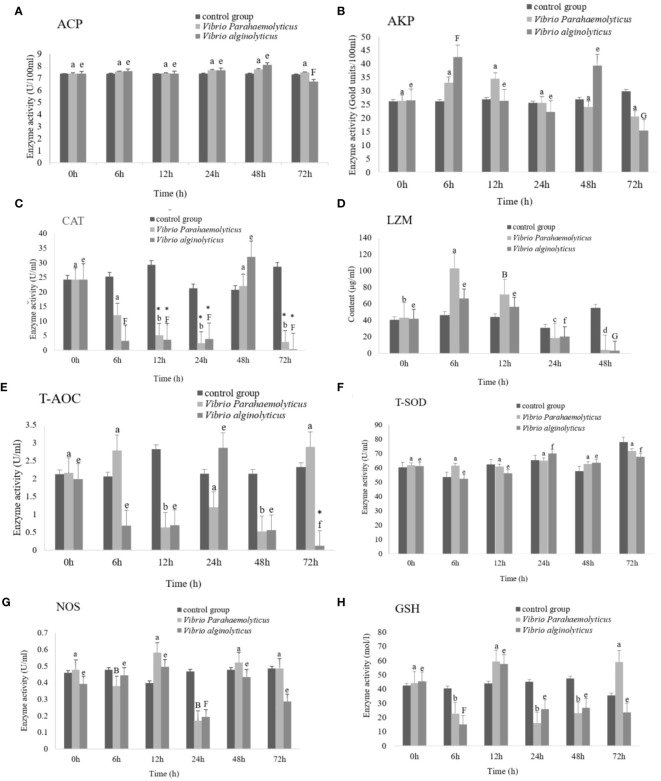
Activities of antioxidant enzymes and immune-related enzymes in hemolymph of *Haliotis discus hannai* under *Vibrio parahaemolyticus* and *Vibrio alginolyticus* stress.

The activities of antioxidant enzymes and immune related enzymes in hemolymph of *H. discus hannai* after *V. parahaemolyticus* and *V. alginolyticus* infection were detected. The experimental results were analysed by double sample t-test. The experiment was divided into 0 h, 6 h, 12 h, 48 h and 72 h, including the following enzymes: superoxide dismutase (T-SOD), lysozyme (LZM), nitric oxide synthase (NOS), total antioxidant capacity (T-AOC), glutathione peroxidase (GSH-Px), catalase (CAT), acid phosphatase (ACP) and alkaline phosphatase (AKP). After *V. alginolyticus* infection, comparing the experimental group with the control group, the 72h experimental group of T-AOC showed significant changes; The 12h, 24h, 48h and 72h experimental groups of CAT had significant differences; There were significant differences in T-SOD at 24h, 48h and 72h; The 48h experimental group of LZM showed significant changes. After *V. parahaemolyticus* infection, AKP activity and LZM content increased first and then decreased, T-SOD activity increased first and then decreased, and T-AOC increased first and then decreased. The activity of NOS and GSH-Px first decreased and then increased, then decreased and then increased. CAT activity first decreased, then increased, then decreased. There was no significant change in ACP. It is speculated that T-AOC, T-SOD, LZM, and CAT can be used as the better disease indicator enzymes for abalone infection with Vibrio (**Figure 14**).

## 4 Discussion

Disease resistance is one of the most essential characteristics of aquaculture species since it is closely related to final yield (32). Haemocytes have critical roles in both invertebrate and vertebrate innate immune defence responses (33, 34). Phagocytosis is an innate immunological response, and the molecular mechanisms of phagocytosis in mammals have been extensively investigated. However, research in this field in invertebrates has been hampered by variable haemocyte subpopulations, a lack of adequate phagocytic lines, and a lack of particular cell markers (35). Flow cytometry, electron microscopy and transcriptome analysis were used in this work to investigate the immune responses of haemocyte in the marine mollusk Pacific abalone *H. discus hannai*. The strategy is a critical step in the investigation of haemocytes in invertebrates.

### 4.1 Transmission electron microscopy to study the phagocytic process

In this study, transmission electron microscopy was used. Phagocytosis is an actin-dependent endocytic process that involves the receptor-mediated engulfment of big particles (diameter *≥* 0.5 µm) into the plasma membrane or plasma membrane-derived vacuoles called phagosomes (36). Phagocytosis is a kind of cellular immunity evoked by foreign particles or pathogen infection, and invertebrate phagocytic haemocytes are thought to be the equivalent of vertebrate macrophages (37). The phagocytic process is thought to consist of two steps, the first of which involves the attachment of foreign particles to the cell surface and the second of which involves internalisation into the cytoplasm, resulting in the engulfment of foreign particles into the cell and the subsequent formation of phagosomes to stimulate microbial digestion (38). Tuan et al. (39, 40) have extensively researched this process. Davids and Yoshino (41) defined haemocyte adhesion as the initial stage in the phagocytosis of foreign particles and migration toward an inflamed location. Images of internalised and engulfed *V. parahaemolyticus*, *S. aureus*, and *V. alginolyticus* were produced in this investigation, as were pseudopodia-adherent bacteria and the subsequent formation of phagosomes. The findings of a bacterium challenge utilising electron microscopy on haemocytes revealed a process during phagosome development that included probing, cup formation, pseudopod expansion, and phagosome formation. Cup-like membrane extensions that are employed to internalise big particles regulated phagocytosis (42). Granulocytes have a larger phagocytic capability than hyalinocytes in general (43). However, in this study, the presence of pseudopodia and changing morphology in hyalinocytes after *V. alginolyticus* stress adds to the evidence that hyalinocytes are the primary haemocytes involved in phagocytising foreign particles (44). The findings might imply that hyalinocytes primarily phagocytose via pseudopodia after *V. alginolyticus* stress.

### 4.2 Identification of differentially expressed genes involved in phagocytosis

Rab proteins are crucial molecules for phagosome activity, and multiple Rab proteins have been identified from the early stage of phagosome formation to the regulation of phagosome maturation (45). In this study, the changes of rho-related protein racA-like and rho GTPase-activating protein 6-like isoform X2 in transcriptome analysis were observed (**Supplementary Table 1**). Furthermore, the actin cytoskeleton’s reorganisation causes pseudopods to form, encircling the foreign particles and sealing the phagocytic cup. This process causes the foreign particles to be internalised into an early phagosome (46). Actin, clone 403-like was discovered in this investigation (**Supplementary Table 1**). The findings demonstrated a degree of consistency between the phagocytic process as shown by electron microscopy and the genes discovered by transcriptome analysis, indicating that the formation and maturation processes of *H. discus hannai* phagosomes were comparable to those observed in vertebrates. This research revealed that phagocytosis was a highly ancient and converted immune system strategy.

### 4.3 Phagocytic activity of haemocytes using flow cytometry

One strong and well-established method for analysing the phagocytic capacity of haemocytes is flow cytometry. It has been applied extensively. Since latex beads are inert polystyrene particles, using them prevents the needless activation of mollusk haemocytes during phagocytosis. Fluorescent microspheres are durable and recognizable (47, 48). The phagocytic rate (the phagocytic percentage, PP) of fluorescent microspheres was higher at 0.5 h, which was 14.35%. The greatest increase in PP of *E. coli* occurred at 1 h (**Figure 4A**). The PP of *S. aureus* was higher than the other five foreign particles, which was about 63%. The PP of *V. harveyi* was about 43%. The greatest increase in PP of *V. parahaemolyticus* occurred at 1.5 h, and decreased at 2.5 h. The PP peak of *V. alginolyticus* in haemocyte was 63.7% at 1.5 h.

The quantitative results of phagocytosis efficacy against six distinct phagocytic mediators were considerably diverse. Many factors, including relative haemocyte size, the properties of foreign particles, the diameter of foreign particles, the pathogenicity of foreign particles, the environment and stress, are likely to contribute to the difference (49). *Scrobicularia plana* haemocytes, for example, could phagocytose three distinct kinds of heat-killed bacteria but not zymosan (50). Furthermore, various haemocyte subpopulations appear to have different preferences in the phagocytosis of different bacteria or viruses. Semi-granular cells, for example, swallowed the majority of white spot syndrome virus (WSSV), whereas in red claw crayfish *Cherax quadricarinatus*, *E. coli* was mostly ingested and eliminated by granular and semi-granular cells (51). The findings suggested that haemocytes may have various phagocytic processes in response to different phagocytic mediators.

### 4.4 Identification of differentially expressed genes involved in immunity

Rho family small GTPases act as molecular switches that regulate a wide range of cellular processes, including cytoskeleton-related events and gene transcription. Rho regulates the formation of actin stress fibers. Rho GTPase-activating proteins (Rho GAPs) are one of the primary groups of Rho GTPase regulators present in all eukaryotes and play important roles in cell cytoskeletal structure, growth, differentiation, neural development and synaptic activities (52). Rho GTPase-activating protein 6 was initially characterised for its rho GAP function, nonenzymatic functions for rho GTPase-activating protein 6 have been identified, including activity as a cytoskeletal protein to promote actin remodelling (53). In this study, in comparing C and A48 group, rho GTPase-activating protein 6-like isoform X2 was significantly down-regulated in A48.

Cell division cycle (Cdc42) and Rac have been shown to activate the p38 MAP kinase and JNK pathways in a variety of cell types. Rac regulates the NADPH oxidase enzyme complex in phagocytic cells (54). Rac A and Cdc42 proteins are members of the Ras superfamily’s Rho family. RacA and Cdc42 are essential regulators of the actin cytoskeleton in eukaryotic cells (54, 55). RacA and Cdc42 are homologous genes that regulate various cellular functions, including cell movement, proliferation, apoptosis, and maintenance of cell morphology and polarity growth (56, 57). In some aspects, Rac and Cdc42 have some overlapping functions, but they are not the same. Rac regulates actin polymerisation at the cell periphery via the Arp2/3 complex, resulting in lamellipodia and membrane ruffles, whereas Cdc42 initiates filopodia formation. Rac and Cdc42 also regulate enzyme activity, such as regulating lipid differentiation and ROS production (58). In this study, in comparing C and A48 group, RacA was significantly up-regulated in A48, in addition, the presence of multiple long and thin cytoplasmic protrusions called filopodia distinguished these haemocytes after bacteria challenge by transmission electron microscopy (**Figure 3**), the result showed that RacA is involved in the formation of pseudopodia.

Cellular immunity to collagen and laminin has been examined in scleroderma (59). In this study, collagen alpha-1(V) chain-like isoform X1 was found to be up-regulated in P48 when compared to C. The data showed that laminin and collagen play key roles in phagocytosis by haemocytes in *H. discus hannai*.

Ca^2+^ is involved in many invertebrate physiological functions and is the principal cation for shell structures. It functions as a second messenger to regulate muscular contraction, neural activation, cell differentiation and cell death. To mediate intracellular Ca^2+^ and Ca^2+^ signals, several energy-dependent Ca^2+^ transporters and Ca^2+^ channels exist (60). Calmodulin, myosin light chain and troponin C are notable for their ability to maintain intracellular Ca^2+^ homeostasis as well as convey Ca^2+^ signals to regulate particular target proteins (60). In this study, compared to the control group, calmodulin was down-regulated in P48 and A48, myosin light chain troponin C and troponin I were down-regulated in A48.

Calmodulin is essential in calcium-dependent signal transduction pathways. Calmodulin is a Ca^2+^-dependent protein with a molecular weight of about 17 kDa that is widely expressed in plant and animal eukaryotic cells (61). It has EF-hand calcium-binding motifs and regulates various physiological processes such as inflammation, stress response, Ca^2+^ homeostasis and apoptosis by interacting with targets such as enzymes and cytoskeletal proteins (62, 63). Calmodulin proteins have been found in mollusks such as Pacific oyster *Crassostrea gigas* and pearl oyster *Pinctada fucata* (64, 65), abalones *H. diversicolor* and *Haliotis discus* (66, 67). Calmodulins are multifunctional proteins that have a role in numerous physiological processes in crustaceans. For example, bacterial stress significantly increased the expression of *Eriocheir sinensis* calmodulin (68). Similarly, the expression level of calmodulin was induced dramatically after *Litopenaeus vannamei* challenged with *V. parahaemolyticus* or White Spot Syndrome Virus (69), which was similar with our findings.

Troponin C interacts with troponin I and troponin T to generate a troponin complex that has a role in muscle contraction regulation (70, 71). Troponin C (TnC) belongs to the EF-hand superfamily. This gene has been found in several species, and its roles have been explained. The findings of Zhao et al. (72) show that TnC participate in *Scylla paramamosain* innate immunity and may play a different function in antiviral and antibacterial immune responses. The function of Troponin T (TnT) in the mud crab *S. paramamosain* was investigated, the results indicated a regulatory role of TnT in the innate immune response of *S. paramamosain* to pathogens (73). The finding reveals a more potential function of TnC and TnT, provides an initial basis for further research into the role of TnC in the innate immunity of invertebrates. In this study, troponin C and troponin I were down-regulated in A48, the results indicated a regulatory role of troponin C and troponin I in the immune response of *H. discus hannai* to *V. alginolyticus*.

Myosins, one of the most significant actin binding proteins, are part of a diverse superfamily of actin-based motors that includes at least 19 different classes (74). Myosin is a hexameric protein composed of two heavy chains (MHC) with a molecular weight of about 200,000 and four light chains (MLC) with a molecular weight of approximately 20,000 (75). Myosin is involved in a variety of processes, including cell signalling, contractility, vesicle trafficking, endocytosis and protein/RNA localisation (76, 77). Myosin light chain protein regulates a variety of processes involved in material transport, muscle shrinkage, and cell division (78).

Myosins are cell skeleton proteins that interact with actin thin filaments (79). Myosin is an actin-dependent molecular motor that moves along actin filaments and generates force by using the energy of adenosine triphosphatase hydrolysis. According to the findings of Han et al. (80), myosin light chain protein was implicated in the regulation of shrimp hemocytic phagocytosis. MYL9 (myosin light chain 9) may regulate muscle contraction by regulating ATPase activity in the myosin head. It controls cytoskeletal dynamics by binding to actin filaments and is therefore involved in cell shape formation, migration, polarity, adhesion, and signal-mechanical transduction (81). Pseudopod extension is propelled by actin polymerisation into filaments that press against the membrane and involves coordinated filament nucleation, growth, bundling, and branching. Not unexpectedly, a host of actin modulators have been linked to phagocytosis completion (36). Some conventional myosins have been implicated in cell adhesion and phagocytosis, creating the driving force for phagosome formation and transport during phagocytosis (82, 83). Other unconventional myosins have also been shown to be involved in the process of phagosome closure and formation (84). Liu et al. (85) discovered that Ran GTPase regulates phagocytosis in WSSV-resistant shrimp by interacting directly with myosin (85). In this study, compared to the control group, actin, myosin heavy chain and myosin light chain were down-regulated in A48. The results indicate that pseudopod extension of haemocyte was affected after 48 h infection by *V. alginolyticus*.

The proteasome degradation cascade is connected to the ubiquitin-conjugated E2 enzyme (UBE2). Previous research (86) discovered increased UBE2 expression levels in individuals exposed to pathogenic organisms such as viruses and bacteria. These studies discovered a link between ubiquitination and the activation of gene cascades involved in the immune response (86). In this study, in comparing C and A48 group, E3 ubiquitin-protein ligase Midline-1-like isoform X3 was significantly down-regulated in A48.

CD53 was originally discovered as a cell surface glycoprotein expressed on all immune cells (87). Predictive biochemical tests revealed that this protein crossed the cell membrane four times, classifying it as a tetraspanin (88). The studies show that CD53 plays key roles in the human immune system. The lack of CD53 was the sole detected phenotypic abnormality in neutrophils from members of a family affected by recurrent microbial infections (89). A more recent genome-wide association analysis of patients with increased susceptibility to mycobacterial infection discovered a connection with CD53 mutations (90). The phenotype associated with CD53 deficit in humans is comparable to that of individuals with leukocyte adhesion defect, indicating the notion that CD53 plays a role in immune cell adhesion and trafficking. Crosslinking CD53 on immune cells promotes cell-cell adhesion, perhaps through effects on the β2 integrin LFA-1 (91, 92). Two recent studies examined the effect of CD53 genetic deletion on the adaptive immune system, indicating CD53’s functions in early B cell development (93), and lymphocyte recirculation via L-selectin stabilisation on the lymphocyte cell surface (94). In this study, compared to the control group, leukocyte surface antigen CD53-like was down-regulated in A48.

Interleukin 6 (IL-6) is a multifunctional cytokine that affects cell development and differentiation and is important in immune response and acute phase responses (95). In this study, compared to the control group, interleukin-6 receptor subunit beta-like was up-regulated in A6.

The suppressors of cytokine signalling (SOCS) gene family plays a role in development and immunity by suppressing cytokine signalling pathways. Cytokines are multifunctional chemicals that have roles in cell proliferation, differentiation, repair and inflammatory organism defence (96). One of the most significant feedback regulators is SOCS, which can suppress excessive cytokine signalling, regulate the JAK-STAT transcription pathway and immunological response, and so on (97). Most SOCSs can be generated by external stimuli including germs and viruses (98). The SOCS family members have conserved structures, which are all defined by an N-terminal motif, a central SH2 domain, and a conserved C-terminal domain, also known as the SOCS box (99). In this study, compared to the control group, SOCS box protein 8-like was down-regulated in A48.

Tumor necrosis factor ligand superfamily (TNFSF) is a cytokine family that regulates both innate and acquired immunity (100). TNFSF has a vital function in innate immunity, primarily involving morphological development, cell proliferation, and apoptosis, as well as participating in inflammatory response, cell invasion, angiogenesis, and so on (100). Many researches have indicated that TNFSF from lower vertebrates may assist in the immune response to viral and bacterial infections, much like in mammals (100). In this study, compared to the control group, tumor necrosis factor ligand superfamily member 10-like was down-regulated in A48.

The Matrix metalloproteinase (MMP) gene family regulates the breakdown of Extra Cellular Matrix (ECM) proteins, which are critical for physiological processes such wound healing, stress response and tissue remodelling (101). MMPs are engaged in various tasks associated to self-stabilisation, such as henogenesis, immunity, tissue repair and pathological processes including tumours, infection and fibrosis (101). In this study, compared to the control group, matrix metalloproteinase-19-like was up-regulated in A48.

### 4.5 Immune-related KEGG pathway enrichment analysis associated with DEGs

#### 4.5.1 Thyroid hormone synthesis

Thyroid hormones modulate the immune system in animals. All vertebrates, including fish, rely on thyroid hormones (THs) for growth, development and metabolism. Quesada-García et al. (102), provide initial evidence for the presence of active thyroid signalling in immunological organs and cells of teleosts.

#### 4.5.2 Th17 cell differentiation signalling pathway

T-helper 17 (Th17) lymphocytes are the most prevalent inflammatory cells seen in mammals. Th17 cells are produced from naive CD4+ T cells that express RORγt, STAT3, IL-17 and IL-22 (103). In mammals, Th17 cells are mostly found in the lamina propria of mucosal tissues (104), where they provide antimicrobial functions via IL-17 and IL-22 production to protect the mucosal barrier against pathogen infections.

#### 4.5.3 Focal adhesion

Focal adhesion (FA) is critical for cell adhesion, migration, and antibacterial immunity (105). The FA signalling pathway not only mediates cell attachment to the ECM and growth substratum, but it also regulates different developmental and pathological processes, making it a transitory structure that allows cells to interact with their environment. Currently, FA is widely investigated in animals and has been linked to disease regulation and cell behaviour (106, 107). FA was found to have a significant proinflammatory function in cell fibrosis and growth, and its assembly and deconstruction regulate cell migration and cancer invasion (108).

#### 4.5.4 Circadian entrainment

The circadian clock is an important regulator because it keeps biological processes and behaviour in check. The clock mechanism has long been understood to play an important function in physiology. This biological system also regulates diurnal immune response oscillations during infections (109). Recent studies (110, 111) indicate that it plays a crucial role in immunological regulation. Many specific immune processes and features are rhythmic, for example leukocyte recruitment, phagocytosis and cytokine production (110–112). The number of circulating leukocytes, cytokine, and chemokine levels fluctuate on a daily oscillations, regulating both the innate and adaptive immune responses. This allows organisms to anticipate daily behavioral changes, lowering the risk of infection and tissue damage (113). Circadian activity of the host immune response, as well as differences in pathogen virulence between day and night, result in a variable risk of infection at certain times of day (110). The circadian clock regulates the diverse manifestations of immunological activity in fish, allowing them to efficiently eliminate pathogens and recover optimally from infection or damage (110).

After *V. alginolyticus* challenge, in comparing C and A6 group, the thyroid hormone synthesis, haematopoietic cell lineage, Th17 cell differentiation signalling pathway had significant difference in A6 (*p* < 0.05) (**Figure 11**). In comparing C and A48 group, regulation of actin cytoskeleton, p53 signalling pathway, focal adhesion, thyroid hormone signalling pathway, circadian entrainment, inflammatory mediator regulation of TRP channels, leukocyte transendothelial migration, Rap1 signalling pathway, Ras signalling pathway and calcium signalling pathway had significant difference in A48 (*p* < 0.05) (**Figure 11**). After *V. parahaemolyticus* challenge, in comparing C and P6, C and P48, thyroid hormone synthesis signalling pathway had significant difference in P6 and P48 (**Figure 11**). These immune-related KEGG pathway enrichment showed that related genes changed after infection with bacteria.

### 4.6 Predicted interactions biomarkers of identified DEGs from STEM

Rab proteins are the primary node in the cell that regulates organelle trafficking, including phagosome trafficking. Rab proteins are essential for phagosome function, and several Rab proteins have been found, ranging from the early stages of phagosome formation to the regulation of phagosome maturation (114). Rab proteins were found to have some of the most noticeable protein expression differences in our transcriptome analysis, and members of the Rab protein family (RABF1-like, Rab-8A-like, Rab-9A, Rab-14, Rab-30-like, Rab-35-like, Rab-37, Rab-43-like, rab5 GDP/GTP exchange factor, RABF1-like, rab3 GTPase-activating protein) were identified. Cathepsins are essential proteins that are activated exclusively at low pH levels and are necessary for the destruction of phagocytosed particles in phagosomes. As a result, the late phagosome becomes more acidic and destructive by increasing V-ATPase accumulation (46). Cathepsin L, cathepsin Z-like, cathepsin B-like and cathepsin O-like exhibited significant differences across groups in this investigation.

Stressors cause a variety of stress responses at the cellular level. Heat shock protein (HSP) expression is a critical biological mechanism for adjusting to environmental changes (115). HSPs are divided into four types based on their molecular weight: small HSPs (15–40 kDa), HSP60, HSP70 (68–80 kDa), and HSP90 (83–99 kDa) (116). HSP gene expression may promote bivalve adaptation to adverse situations by boosting HSP expression (117, 118). Changes in HSP gene expression levels have showed promise in reducing different abiotic and pathogenic biotic stresses in aquatic animals (119). Hsp20, heat shock factor protein 1, heat shock 70 kDa protein, heat shock protein 60A-like and 90 kDa heat shock protein were discovered in this study.

Superoxide dismutase (SOD) and catalase (CAT) are recognised to be important in protecting cells from reactive oxygen species (ROS). SOD and CAT are essential for reducing oxidative stress and maintaining fish homeostasis. When the fish are subjected to stressors, they successfully scavenge reactive oxygen species (ROS) (120). This paradoxical rise in ROS generation and development of oxidative stress in fish and other species has also been described (121). Extracellular superoxide dismutase [Cu-Zn]-like isoform X3 and catalase were shown to be down-regulated throughout the process in the C, A6, and A48 groups.

These HSPs are critical for stress management because they promote antioxidant systems and immunological responses (120). The generation of lipoperoxides by ROS was discovered to have an effect on the structure and chaperone function of HSP70. Furthermore, several HSP70 members have been shown to function as redox sensors, sending redox signals (122). As an ATP-dependent enzyme, HSP70 is essential. Furthermore, HSP70 signalling has been shown to increase the expression of antioxidant enzymes, including SOD (123).

Furthermore, after a *V. alginolyticus* challenge, alkaline phosphatase-like were up-regulated in the P6 group. Histone, interferon, mucin-like and toll-like receptors have all been discovered. In reaction to stress, these genes can be employed as innate immune markers. As a result, biomarkers linked with *V. alginolyticus* and *V. parahaemolyticus* stress may be used to measure abalone physiological state.

### 4.7 Phagocytosis capacities of the haemocytes

In molluscs, there is continuous debate concerning haemocytes’ phagocytic capability. Previous research demonstrated that both granulocytes and hyalinocytes were immune effector cells. Molluscan granulocytes are often more active than hyalinocytes (124). However, some researches have found that hyalinocytes have more oxidative activity than granulocytes (125). According to Donaghy et al.’ study (126), hyalinocytes have a stronger phagocytic potential than granulocytes in the Suminoe oyster, *Crassostrea ariakensis*.

Hyalinocytes were the sole haemocytes involved in latex bead phagocytosis *in vitro* in the tiger shrimp *Penaeus monodon* (127). In crayfish, hyalinocytes were the primary haemocytes involved in phagocytosis. According to Johansson et al. ‘ study (128), granulocytes were primarily engaged in cytotoxicity as well as the storage and release of the proPO system. *Eriocheir sinensis* has a higher proportion of hyalinocytes than granulocytes or semigranulocytes, which might be explained by its higher phagocytic ratio (44). When haemocytes were temporarily cultured *in vitro*, the primary phagocytic cells in the phagocytosis of Zymosan (3 μm) and yeast (6 μm) were the hyalinocytes of *Carcinus aestuarii* and *Macrobrachium rosenbergii*; however, when *M. rosenbergii*, *Penaeus paulensis*, and *Macrobrachium acanthurus* were incubated with the yeast *Saccharmyces cerevisiae* for one hour, semigranulocytes and granulocytes were observed to be more actively phagocytic in these three species (129–131). In this study, electron microscopy revealed a process of phagosome formation in haemocytes challenged with *V. parahaemolyticus*, *V. alginolyticus* and *S. aureus*. The findings indicate that hyalinocytes may play key roles in the immunological response in *H. discus hanna*i after *V. alginolyticus* stress.

## 5 Conclusions

In the present study, we demonstrated that electron microscopy coupled with flow cytometry and transcriptome analysis resulted in a comprehensive knowledge of haemocyte immune responses in *H. discus hannai*. Electron microscopy data for hyalinocytes revealed a process of phagosome formation in response to *V. parahaemolyticus*, *V. alginolyticus*, and *S. aureus* challenge. Quantitative transcriptome analysis found a series of differences in gene expression during phagosome formation and maturation, including Rab proteins, actin-related gene, rho GTPase-activating protein 6-like isoform X2, RacA, collagen alpha-1(V) chain-like isoform X1, calmodulin, troponin I, troponin C, myosin light chain, myosin heavy chain, leukocyte surface antigen CD53, interleukin 6 (IL-6), suppressors of cytokine signalling (SOCS), tumor necrosis factor ligand superfamily (TNFSF), matrix metalloproteinase (MMP). In response to *V. parahaemolyticus* or *V. alginolyticus* exposure, the findings show that hyalinocytes play a role in the cellular immune response of *H. discus hannai* after *V. alginolyticus* stress. The specific roles of these haemocytes deserve additional exploration.

## Data availability statement

The original contributions presented in the study are publicly available. This data can be found here: https://www.ncbi.nlm.nih. gov/geo/under the accession number PRJNA1117375.

## Ethics statement

The animal study was reviewed and approved by the Institutional Animal Care and Use Committee (IACUC) of the Fujian Agriculture and Forestry University for the Ethics of Animal Experiments.

## Author contributions

ZM: Methodology, Writing – original draft, Data curation. YW: Methodology, Writing – original draft, Investigation. YZ: Writing – original draft. WZ: Methodology, Writing – review & editing, Data curation. MJ: Writing – original draft. XS: Writing – review & editing. HW: Writing – review & editing. XC: Writing – review & editing. GD: Formal analysis, Methodology, Writing – original draft.
