# Supplementary material for: Morphologic, cytometric, quantitative transcriptomic and functional characterisation provide insights into the haemocyte immune responses of Pacific abalone (Haliotis discus hannai)
Source: Front Immunol. 2024 Jul 2;15:1376911. doi: 10.3389/fimmu.2024.1376911 (PMC11250055; doi:10.3389/fimmu.2024.1376911)
Supplement: Supplementary file 1 [file Table_1.docx]

**Supplementary Table 1**. Differential genes comparison between five samples

| Id | C_mean | mean | log2(fc) | PValue | FDR |  | Symbol | Description |
| --- | --- | --- | --- | --- | --- | --- | --- | --- |
| **C vs A6** | **C** | **A6** |  |  |  |  |  |  |
| Unigene0003336 | 0.065 | 10.57 | 7.345319943 | 2.78E-06 | 0.015880981 | Up | prtgb | XP_046331545.1 interleukin-6 receptor subunit beta-like [*Haliotis rufescens*] |
| Unigene0001085 | 0.001 | 15.88 | 13.95492329 | 7.22E-06 | 0.027929537 | Up | -- | XP_046336404.1 protein turtle homolog B-like [*Haliotis rufescens*] |
| **C vs A48** |  |  |  |  |  |  |  |  |
|  | **C** | **A48** |  |  |  |  |  |  |
| Unigene0039266 | 38.88 | 0.001 | -15.2467406 | 5.96E-07 | 0.001144466 | Down | bdh2 | XP_046363821.1 3-hydroxybutyrate dehydrogenase type 2-like [*Haliotis rufescens*] |
| Unigene0044567 | 26.41 | 0.001 | -14.68879668 | 0.000107991 | 0.032682271 | Down | PIN | XP_046376470.1 con-Ins Im2-like [*Haliotis rufescens*] |
| Unigene0081566 | 25.85 | 0.001 | -14.65787666 | 7.01E-07 | 0.001282358 | Down | MID1 | XP_046336009.1 E3 ubiquitin-protein ligase Midline-1-like isoform X3 [*Haliotis rufescens*] |
| Unigene0043541 | 9700.845 | 225.59 | -5.426335398 | 1.42E-06 | 0.002024427 | Down | F46H5.3 | P51544.1 RecName: Full=Arginine kinase; Short=AK [*Haliotis madaka*] |
| Unigene0042150 | 22.27 | 0.03 | -9.535923342 | 1.83E-05 | 0.010186671 | Down | Arhgap6 | XP_046369427.1 rho GTPase-activating protein 6-like isoform X2 [*Haliotis rufescens*] |
| Unigene0002677 | 1.955 | 89.875 | 5.522679353 | 0.000167246 | 0.044639575 | Up | racA | XP_046357585.1 rho-related protein racA-like [*Haliotis rufescens*] |
| Unigene0061241 | 23.495 | 0.305 | -6.267400715 | 0.000112678 | 0.033571974 | Down | Tspan4 | XP_046366831.1 leukocyte surface antigen CD53-like [*Haliotis rufescens*] |
| Unigene0001551 | 34.83 | 0.395 | -6.462334009 | 3.51E-05 | 0.014351856 | Down | TKFC | XP_046370708.1 triokinase/FMN cyclase-like isoform X1 [*Haliotis rufescens*] |
| Unigene0036036 | 24.61 | 0.001 | -14.58695704 | 4.46E-05 | 0.016513725 | Down | -- | XP_046325743.1 multiple epidermal growth factor-like domains protein 11 isoform X2 [*Haliotis rufescens*] |
| Unigene0064733 | 3412.245 | 104.175 | -5.033640231 | 6.32E-06 | 0.005594325 | Down | PMY | BAJ61596.1 paramyosin [*Haliotis discus discus*] |
| Unigene0057157 | 13408.73 | 475.445 | -4.817750331 | 8.14E-06 | 0.00634568 | Down | CNN3 | XP_046329182.1 calponin-1-like [*Haliotis rufescens*] |
| Unigene0099728 | 327.68 | 12.48 | -4.714597781 | 0.000152642 | 0.041608372 | Down | -- | XP_046360725.1 calponin homology domain-containing protein DDB_G0272472-like isoform X1 [*Haliotis rufescens*] |
| Unigene0090985 | 79.2 | 0.09 | -9.781359714 | 4.09E-06 | 0.004496593 | Down | CPK15 | XP_046326461.1 calmodulin-like [*Haliotis rufescens*] |
| Unigene0069977 | 23.235 | 0.001 | -14.50401202 | 1.33E-06 | 0.001962244 | Down | NOX5 | XP_046368239.1 NADPH oxidase 5-like isoform X2 [*Haliotis rufescens*] |
| Unigene0095003 | 23.94 | 0.001 | -14.54713553 | 8.40E-05 | 0.02694241 | Down | Hdhd5 | XP_046342675.1 haloacid dehalogenase-like hydrolase domain-containing 5 isoform X1 [*Haliotis rufescens*] |
| Unigene0032249 | 32.99 | 0.001 | -15.00974116 | 1.39E-05 | 0.008637105 | Down | -- | UFT26661.1 myomodulin prepropeptide [*Haliotis discus hannai*] |
| Unigene0055305 | 5035.835 | 198.17 | -4.667420532 | 3.48E-05 | 0.014351856 | Down | act-1 | XP_041046298.1 actin, clone 403-like [*Carcharodon carcharias*] |
| Unigene0039205 | 60.025 | 0.03 | -10.96638528 | 1.67E-07 | 0.000852264 | Down | -- | XP_046381436.1 profilin-like [*Haliotis rufescens*] |
| Unigene0045511 | 1195.82 | 37.46 | -4.996505535 | 3.73E-05 | 0.014932466 | Down | -- | XP_046364861.1 transgelin-2-like isoform X1 [*Haliotis rufescens*] |
| Unigene0090135 | 958.265 | 23.905 | -5.325040365 | 6.05E-06 | 0.005532926 | Down | SM20 | ARR97158.1 troponin C [*Haliotis discus discus*] |
| Unigene0037904 | 5083.18 | 303.65 | -4.065250045 | 0.000133806 | 0.038802878 | Down | tni-4 | XP_046367618.1 troponin I-like isoform X4 [*Haliotis rufescens*] |
| Unigene0077101 | 5152.2 | 193.625 | -4.733851453 | 1.89E-05 | 0.010215172 | Down | up | XP_046378789.1 troponin T-like isoform X18 [*Haliotis rufescens*] |
| Unigene0072093 | 17.355 | 0.001 | -14.08306374 | 1.60E-05 | 0.009738483 | Down | TTN | XP_046351780.1 titin-like isoform X3 [*Haliotis rufescens*] |
| Unigene0027310 | 64.315 | 1.995 | -5.010694601 | 6.90E-05 | 0.022853857 | Down | sls | XP_046364053.1 titin-like isoform X5 [*Haliotis rufescens*] |
| Unigene0027616 | 13905.26 | 584.37 | -4.572604794 | 2.21E-05 | 0.010641209 | Down | mlc-3 | XP_046367940.1 myosin essential light chain, striated adductor muscle-like [*Haliotis rufescens*] |
| Unigene0056858 | 3724.7 | 160.495 | -4.536523973 | 3.99E-05 | 0.015472325 | Down | MYL5 | XP_046327497.1 myosin regulatory light chain, smooth muscle-like [*Haliotis rufescens*] |
| Unigene0060888 | 73.25 | 0.93 | -6.299454233 | 9.27E-05 | 0.028724695 | Down | sqh | XP_046331186.1 myosin regulatory light chain 12A-like [*Haliotis rufescens*] |
| Unigene0030554 | 1527.61 | 35.42 | -5.430568247 | 1.74E-06 | 0.002382059 | Down | MYH7 | XP_046351341.1 myosin heavy chain, striated muscle-like [*Haliotis rufescens*] |
| Unigene0004520 | 13.855 | 0.001 | -13.75811909 | 1.34E-05 | 0.008413676 | Down | MYH16 | XP_046351835.1 myosin heavy chain, striated muscle-like [*Haliotis rufescens*] |
| Unigene0030552 | 488.255 | 23.12 | -4.400421515 | 0.000107182 | 0.032682271 | Down | PARA | XP_046351176.1 myosin heavy chain, striated muscle-like isoform X10 [*Haliotis rufescens*] |
| Unigene0014552 | 1119.145 | 31.845 | -5.135186286 | 1.65E-05 | 0.009765868 | Down | Mlp84B | XP_046332524.1 muscle LIM protein Mlp84B-like [*Haliotis rufescens*] |
| Unigene0090942 | 64.505 | 4134.465 | 6.002145853 | 3.77E-07 | 0.000905935 | Up | Mmp19 | XP_046333725.1 matrix metalloproteinase-19-like [*Haliotis rufescens*] |
| Unigene0062444 | 87.96 | 0.58 | -7.244650894 | 2.02E-05 | 0.010469309 | Down | METTL7A | XP_046369826.1 methyltransferase-like protein 7A [*Haliotis rufescens*] |
| Unigene0045460 | 56.865 | 1.245 | -5.51332331 | 6.27E-05 | 0.021319727 | Down | -- | XP_041370407.1 ankyrin repeat and SOCS box protein 8-like [*Gigantopelta aegis*] |
| Unigene0035336 | 11.99 | 0.001 | -13.54954404 | 8.75E-06 | 0.00634568 | Down | Magi1 | XP_046353264.1 membrane-associated guanylate kinase, WW and PDZ domain-containing protein 1-like isoform X5 [*Haliotis rufescens*] |
| Unigene0071794 | 37.195 | 0.03 | -10.27593048 | 2.85E-06 | 0.003423436 | Down | Gld | XP_046378592.1 glucose dehydrogenase [FAD, quinone]-like isoform X1 [*Haliotis rufescens*] |
| Unigene0053117 | 2515.68 | 81.86 | -4.941645943 | 1.74E-05 | 0.009850588 | Down | Tagln2 | XP_046376465.1 myophilin-like [*Haliotis rufescens*] |
| Unigene0055592 | 0.001 | 20.23 | 14.3042087 | 2.59E-07 | 0.000852264 | Up | Kif28p | XP_046369604.1 kinesin-like protein KIF28P isoform X3 [*Haliotis rufescens*] |
| Unigene0074598 | 26.27 | 0.015 | -10.77423798 | 4.39E-07 | 0.000937347 | Down | Spon1 | XP_046360194.1 somatomedin-B and thrombospondin type-1 domain-containing protein-like isoform X1 [*Haliotis rufescens*] |
| Unigene0098709 | 66.86 | 0.001 | -16.02885573 | 1.15E-05 | 0.007620121 | Down | -- | XP_046374238.1 growth arrest and DNA damage-inducible proteins-interacting protein 1-like [*Haliotis rufescens*] |
| Unigene0103925 | 25.945 | 0.001 | -14.66316892 | 0.00019876 | 0.04993023 | Down | -- | XP_046378817.1 prothoracicostatic peptide-like [*Haliotis rufescens*] |
| Unigene0080858 | 1.24 | 1377.065 | 10.11704082 | 0.000140593 | 0.039157117 | Up | -- | XP_046382097.1 hemagglutinin/amebocyte aggregation factor-like [*Haliotis rufescens*] |
| Unigene0077316 | 13.395 | 0.001 | -13.70940696 | 7.83E-06 | 0.006270238 | Down | CD40LG | XP_046325980.1 tumor necrosis factor ligand superfamily member 10-like [*Haliotis rufescens*] |
|  |  |  |  |  |  |  |  |  |
| **C vs P6** | **C** | **P6** |  |  |  |  |  |  |
| Unigene0030554 | 1527.61 | 111.28 | -3.779010039 | 1.19E-05 | 0.017566086 | Down | MYH7 | XP_046351341.1 myosin heavy chain, striated muscle-like [*Haliotis rufescens*] |
| Unigene0063050 | 8.27 | 0.001 | -13.01367161 | 2.73E-05 | 0.029812077 | Down | -- | XP_046380955.1 supervillin-like isoform X4 [*Haliotis rufescens*] |
| Unigene0032792 | 66.725 | 0.715 | -6.544140348 | 1.45E-07 | 0.001043119 | Down | TM_0325 | XP_046365267.1 uncharacterized oxidoreductase MexAM1_META1p0182-like [*Haliotis rufescens*] |
| Unigene0080858 | 1.24 | 754.57 | 9.249170812 | 8.92E-13 | 4.10E-08 | Up | -- | XP_046382097.1 hemagglutinin/amebocyte aggregation factor-like [*Haliotis rufescens*] |
| Unigene0043496 | 31.625 | 0.725 | -5.446940674 | 4.86E-06 | 0.008932772 | Down | -- | XP_046366779.1 allatostatin-A receptor-like isoform X2 [*Haliotis rufescens*] |
| Unigene0071065 | 26.08 | 0.001 | -14.67065625 | 1.25E-05 | 0.018010832 | Down | nAChRalpha1 | ABU51880.1 acetylcholine binding protein 1 [*Haliotis discus hannai*] |
| Unigene0080610 | 0.575 | 33.83 | 5.878597411 | 3.03E-05 | 0.031599369 | Up | Oacyl | XP_046340240.1 O-acyltransferase like protein-like [*Haliotis rufescens*] |
| **C vs P48** |  |  |  |  |  |  |  |  |
|  | **C** | **P48** |  |  |  |  |  |  |
| Unigene0053302 | 46.57 | 0.53 | -6.457264712 | 2.92E-05 | 0.036385854 | Down | COL11A1 | XP_046380609.1 collagen alpha-1(V) chain-like isoform X1 [*Haliotis rufescens*] |
| Unigene0098416 | 0.115 | 49.41 | 8.747025384 | 1.07E-05 | 0.020077189 | Up | ALB | NP_000468.1 albumin preproprotein [*Homo sapiens*] |
| Unigene0090985 | 79.2 | 0.125 | -9.307428525 | 2.70E-05 | 0.036385854 | Down | CPK15 | XP_046326461.1 calmodulin-like [*Haliotis rufescens*] |
| Unigene0085652 | 166.94 | 2.52 | -6.049762132 | 1.84E-05 | 0.030394442 | Down | -- | XP_046335363.1 kyphoscoliosis peptidase-like [*Haliotis rufescens*] |
