# Supplementary material for: Morphologic, cytometric, quantitative transcriptomic and functional characterisation provide insights into the haemocyte immune responses of Pacific abalone (Haliotis discus hannai)
Source: Front Immunol. 2024 Jul 2;15:1376911. doi: 10.3389/fimmu.2024.1376911 (PMC11250055; doi:10.3389/fimmu.2024.1376911)
Supplement: Supplementary file 2 [file Table_2.docx]

**Supplementary Table 2**. Gene Ontology (GO) enrichment analysis of differentially expressed genes

| ID | C _fpkm | P48 _fpkm | P6 _fpkm | A48 _fpkm | A6 _fpkm | Symbol | Description |
| --- | --- | --- | --- | --- | --- | --- | --- |
| Unigene0001551 | 70.95 | 6.27 | 11.22 | 7.17 | 509.78 | TKFC | XP_046370708.1 triokinase/FMN cyclase-like isoform X1 [*Haliotis rufescens*] |
| Unigene0001763 | 135.41 | 4.23 | 55.67 | 19.52 | 115.28 | AKR1 | XP_046346936.1 uncharacterized protein LOC124127577 isoform X1 *[Haliotis rufescens*] |
| Unigene0001763 | 135.41 | 4.23 | 55.67 | 19.52 | 53.46 | AKR1 | XP_046346936.1 uncharacterized protein LOC124127577 isoform X1 [*Haliotis rufescens*] |
| Unigene0002677 | 11.68 | 0.31 | 4.76 | 180.39 | 10.13 | racA | XP_046357585.1 rho-related protein racA-like [*Haliotis rufescens*] |
| Unigene0003336 | 11.7 | 1.39 | 3.53 | 6.83 | 46.08 | prtgb | XP_046331545.1 interleukin-6 receptor subunit beta-like [*Haliotis rufescens*] |
| Unigene0007201 | 359.15 | 6.23 | 379.11 | 42.22 | 213.81 | CBFB | XP_046378861.1 core-binding factor subunit beta-like isoform X2 [*Haliotis rufescens*] |
| Unigene0022529 | 541.75 | 0 | 0 | 0 | 0.92 | HBB | AAD19696.1 hemoglobin beta chain [Homo sapiens] |
| Unigene0029628 | 78.11 | 26.19 | 79.96 | 10.21 | 30.11 | Fhod3 | XP_046364598.1 FH1/FH2 domain-containing protein 3-like [*Haliotis rufescens*] |
| Unigene0030552 | 1030.63 | 288.76 | 1069.78 | 465.16 | 5288.13 | PARA | XP_046351176.1 myosin heavy chain, striated muscle-like isoform X10 [*Haliotis rufescens*] |
| Unigene0031634 | 162.65 | 36.13 | 117.81 | 35.53 | 81.5 | RyR | XP_046359074.1 ryanodine receptor 2-like isoform X17 [*Haliotis rufescens*] |
| Unigene0035336 | 24.94 | 5.89 | 12.07 | 5.08 | 36.28 | Magi1 | XP_046353264.1 membrane-associated guanylate kinase,  WW and PDZ domain-containing protein 1-like isoform X5 [*Haliotis rufescens*] |
| Unigene0037904 | 10701.6 | 3957.11 | 9566.61 | 3747.05 | 3852.24 | tni-4 | XP_046367618.1 troponin I-like isoform X4 [*Haliotis rufescens*] |
| Unigene0039266 | 82.75 | 42.23 | 85.78 | 1.01 | 51.91 | bdh2 | XP_046363821.1 3-hydroxybutyrate dehydrogenase type 2-like [*Haliotis rufescens*] |
| Unigene0042150 | 47.3 | 20.65 | 55.28 | 13.78 | 66.74 | Arhgap6 | XP_046369427.1 rho GTPase-activating protein 6-like isoform X2 [*Haliotis rufescens*] |
| Unigene0044790 | 137.12 | 27.49 | 95.51 | 27.85 | 42.6 | Steap4 | XP_046378683.1 metalloreductase STEAP4-like [*Haliotis rufescens*] |
| Unigene0045497 | 101.63 | 88.05 | 2.66 | 6.17 | 28.85 | FMRFa | XP_046341294.1 FMRF-amide neuropeptides-like [*Haliotis rufescens*] |
| Unigene0053329 | 120.41 | 30.73 | 1.26 | 53.78 | 71.56 | TADA2A | XP_046339003.1 transcriptional adapter 2-alpha-like [*Haliotis rufescens*] |
| Unigene0060107 | 43.04 | 5.1 | 15.81 | 7.41 | 19.19 | PRRX1 | XP_046330244.1 paired mesoderm homeobox protein 2-like [*Haliotis rufescens*] |
| Unigene0060888 | 154.13 | 39.56 | 80.66 | 17.17 | 63.03 | sqh | XP_046331186.1 myosin regulatory light chain 12A-like [*Haliotis rufescens*] |
| Unigene0061241 | 53.06 | 27.09 | 37.37 | 12.42 | 72.9 | Tspan4 | XP_046366831.1 leukocyte surface antigen CD53-like [*Haliotis rufescens*] |
| Unigene0062444 | 182.9 | 47.05 | 252.77 | 69.58 | 142.97 | METTL7A | XP_046369826.1 methyltransferase-like protein 7A [*Haliotis rufescens*] |
| Unigene0065017 | 387.38 | 124.01 | 268.34 | 46.95 | 226.74 | PDLIM1 | XP_046361471.1 PDZ and LIM domain protein Zasp-like isoform X5 [*Haliotis rufescens*] |
| Unigene0066342 | 20.31 | 6.04 | 9.83 | 4.08 | 127.86 | Nmdar1 | XP_046340755.1 glutamate [NMDA] receptor subunit 1-like isoform X3 [*Haliotis rufescens*] |
| Unigene0068201 | 11.53 | 155.92 | 22.16 | 373.77 | 45.01 | SPCP1E11.10 | XP_046344278.1 uncharacterized protein LOC124125011 isoform X1 [*Haliotis rufescens*] |
| Unigene0069048 | 55.63 | 8.95 | 29.09 | 1.75 | 23.09 | tbx1-b | XP_046360211.1 T-box transcription factor TBX1-like [*Haliotis rufescens*] |
| Unigene0069977 | 48.58 | 17.33 | 24.38 | 10.1 | 66.34 | NOX5 | XP_046368239.1 NADPH oxidase 5-like isoform X2 [*Haliotis rufescens*] |
| Unigene0071836 | 45.67 | 97.46 | 158.78 | 585.63 | 31.64 | dvr1 | XP_046365383.1 protein DVR-1-like [*Haliotis rufescens*] |
| Unigene0075614 | 231.38 | 106.87 | 184.33 | 37.56 | 115.98 | Zasp52 | XP_046346939.1 PDZ and LIM domain protein 3-like [*Haliotis rufescens*] |
| Unigene0077316 | 31.3 | 17.39 | 21.73 | 7.81 | 44.38 | CD40LG | XP_046325980.1 tumor necrosis factor ligand superfamily member 10-like [*Haliotis rufescens*] |
| Unigene0081343 | 29.27 | 12.4 | 8.57 | 3.06 | 16.83 | Aoc1 | XP_046377421.1 putative amine oxidase [copper-containing] [*Haliotis rufescens*] |
| Unigene0081566 | 55.23 | 5.26 | 24.39 | 5.28 | 196.71 | MID1 | XP_046336009.1 E3 ubiquitin-protein ligase Midline-1-like isoform X3 [*Haliotis rufescens*] |
| Unigene0096861 | 38.74 | 36.17 | 23.55 | 7.36 | 36.68 | SFRP1 | XP_046330096.1 secreted frizzled-related protein 1-like isoform X1 [*Haliotis rufescens*] |
| Unigene0098416 | 0.23 | 126.94 | 76.31 | 34.26 | 55.31 | ALB | NP_000468.1 albumin preproprotein [*Homo sapiens*] |
| Unigene0098416 | 0.23 | 126.94 | 76.31 | 34.26 | 54.08 | ALB | NP_000468.1 albumin preproprotein [*Homo sapiens*] |
| Unigene0098713 | 0 | 336.93 | 97.17 | 3.04 | 80.22 | Alb | CAA24532.1 unnamed protein product [*Rattus norvegicus*] |
| Unigene0098713 | 0 | 336.93 | 97.17 | 3.04 | 69.13 | Alb | CAA24532.1 unnamed protein product [*Rattus norvegicus*] |
| Unigene0100902 | 9.75 | 24.45 | 8.03 | 36.05 | 45.99 | birc7-a | XP_046379107.1 baculoviral IAP repeat-containing protein 3-like [*Haliotis rufescens*] |
