# Supplementary material for: Morphologic, cytometric, quantitative transcriptomic and functional characterisation provide insights into the haemocyte immune responses of Pacific abalone (Haliotis discus hannai)
Source: Front Immunol. 2024 Jul 2;15:1376911. doi: 10.3389/fimmu.2024.1376911 (PMC11250055; doi:10.3389/fimmu.2024.1376911)
Supplement: Supplementary file 5 [file Table_5.docx]

**Supplementary table. 5. Predicted interactions of identified DEGs from STEM in C, A6 and A48 groups**

| **Symbol** | **Description** |
| --- | --- |
| txn2 | Thioredoxin 2. (166 aa) |
| atg7 | Ubiquitin-like modifier-activating enzyme ATG7; E1-like activating enzyme involved in the 2 ubiquitin-like systems required for cytoplasm to vacuole transport (Cvt) and autophagy. Activates ATG12 for its conjugation with ATG5 as well as the ATG8 family proteins for their conjugation with phosphatidylethanolamine. Both systems are needed for the ATG8 association to Cvt vesicles and autophagosomes membranes. Required for autophagic death induced by caspase-8 inhibition. Required for mitophagy which contributes to regulate mitochondrial quantity and quality by eliminating the mitochondria [...] (711 aa) |
| ecsit | Evolutionarily conserved signaling intermediate in Toll pathway, mitochondrial; Adapter protein of the Toll-like and IL-1 receptor signaling pathway that is involved in the activation of NF-kappa-B. (452 aa) |
| actr2b | Actin-related protein 2-B; ATP-binding component of the Arp2/3 complex, a multiprotein complex that mediates actin polymerization upon stimulation by nucleation-promoting factor (NPF) (By similarity). The Arp2/3 complex mediates the formation of branched actin networks in the cytoplasm, providing the force for cell motility (By similarity). Seems to contact the pointed end of the daughter actin filament (By similarity). In addition to its role in the cytoplasmic cytoskeleton, the Arp2/3 complex also promotes actin polymerization in the nucleus, thereby regulating gene transcription and [...] (394 aa) |
| calr | Calreticulin. (417 aa) |
| LOC100004321 | BCL2-associated X, apoptosis regulator a. (197 aa) |
| actr3 | Actin-related protein 3; Belongs to the actin family. (231 aa) |
| hist2h2l | Histone H2B 3; Core component of nucleosome. Nucleosomes wrap and compact DNA into chromatin, limiting DNA accessibility to the cellular machineries which require DNA as a template. Histones thereby play a central role in transcription regulation, DNA repair, DNA replication and chromosomal stability. DNA accessibility is regulated via a complex set of post-translational modifications of histones, also called histone code, and nucleosome remodeling. (126 aa) |
| setmar | SET domain and mariner transposase fusion protein. (293 aa) |
| tlr4bb | Toll-like receptor 4b, duplicate b. (819 aa) |
| hsp70.3 | Heat shock cognate 70-kd protein, tandem duplicate 3; Belongs to the heat shock protein 70 family. (643 aa) |
| trip10a | Cdc42-interacting protein 4 homolog; Required to coordinate membrane tubulation with reorganization of the actin cytoskeleton during endocytosis. Belongs to the FNBP1 family. (537 aa) |
| tlr2 | Toll-like receptor 2. (788 aa) |
| casp8 | Caspase 8, apoptosis-related cysteine peptidase; Belongs to the peptidase C14A family. (476 aa) |
| LOC798445 | Uncharacterized protein; Belongs to the peptidase C14A family. (184 aa) |
| calm2a | Calmodulin; Calmodulin mediates the control of a large number of enzymes, ion channels and other proteins by Ca(2+). Among the enzymes to be stimulated by the calmodulin-Ca(2+) complex are a number of protein kinases and phosphatases. (149 aa) |
| h2ax | Histone H2AX; Variant histone H2A which replaces conventional H2A in a subset of nucleosomes. Nucleosomes wrap and compact DNA into chromatin, limiting DNA accessibility to the cellular machineries which require DNA as a template. Histones thereby play a central role in transcription regulation, DNA repair, DNA replication and chromosomal stability. DNA accessibility is regulated via a complex set of post- translational modifications of histones, also called histone code, and nucleosome remodeling. Required for checkpoint-mediated arrest of cell cycle progression in response to low dos [...] (142 aa) |
| calml4a | Calmodulin-like 4a. (153 aa) |
| cnn1a | Calponin; Thin filament-associated protein that is implicated in the regulation and modulation of smooth muscle contraction. It is capable of binding to actin, calmodulin, troponin C and tropomyosin. The interaction of calponin with actin inhibits the actomyosin Mg-ATPase activity. (287 aa) |
| zgc:173552 | Histone H3; Belongs to the histone H3 family. (136 aa) |
| cat | Catalase; Occurs in almost all aerobically respiring organisms and serves to protect cells from the toxic effects of hydrogen peroxide; Belongs to the catalase family. (526 aa) |
| capzb | F-actin-capping protein subunit beta; F-actin-capping proteins bind in a Ca(2+)-independent manner to the fast growing ends of actin filaments (barbed end) thereby blocking the exchange of subunits at these ends. Unlike other capping proteins (such as gelsolin and severin), these proteins do not sever actin filaments. (273 aa) |
| txnl1 | Thioredoxin-like 1. (289 aa) |
| pxdn | Peroxidasin. (1460 aa) |
| tlr3 | Toll-like receptor 3; Belongs to the Toll-like receptor family. (903 aa) |
| hyou1 | Hypoxia up-regulated protein 1; Has a pivotal role in cytoprotective cellular mechanisms triggered by oxygen deprivation. May play a role as a molecular chaperone and participate in protein folding; Belongs to the heat shock protein 70 family. (980 aa) |
