# Supplementary material for: Morphologic, cytometric, quantitative transcriptomic and functional characterisation provide insights into the haemocyte immune responses of Pacific abalone (Haliotis discus hannai)
Source: Front Immunol. 2024 Jul 2;15:1376911. doi: 10.3389/fimmu.2024.1376911 (PMC11250055; doi:10.3389/fimmu.2024.1376911)
Supplement: Supplementary file 6 [file Table_6.docx]

**Supplementary table. 6. Predicted interactions of identified DEGs from STEM in C, P6 and P48 groups**

| **Symbol** | **Description** |
| --- | --- |
| actr3 | Actin-related protein 3; Belongs to the actin family. (231 aa) |
| cd63 | Tetraspanin. (237 aa) |
| map2k6 | Dual specificity mitogen-activated protein kinase kinase 6; Dual specificity protein kinase which acts as an essential component of the MAP kinase signal transduction pathway. Catalyzes the concomitant phosphorylation of a threonine and a tyrosine residue in the MAP kinases p38 and plays an important role in the regulation of cellular responses to cytokines and all kinds of stresses. The p38 MAP kinase signal transduction pathway leads to direct activation of transcription factors. Phosphorylation by MAP2K6 asymmetrically activates p38 on one side of the blastodisc, an event which is n [...] (363 aa) |
| nras | GTPase NRas; Ras proteins bind GDP/GTP and possess intrinsic GTPase activity; Belongs to the small GTPase superfamily. Ras family. (188 aa) |
| acta1b | Actin alpha 1, skeletal muscle b. (377 aa) |
| tnfsf10 | Tumor necrosis factor (Ligand) superfamily, member 10 like 2; Belongs to the tumor necrosis factor family. (299 aa) |
| casp21 | Caspase 21, apoptosis-related cysteine peptidase; Belongs to the peptidase C14A family. (266 aa) |
| wsb1 | WD repeat and SOCS box-containing protein 1; Probable substrate-recognition component of a SCF-like ECS (Elongin-Cullin-SOCS-box protein) E3 ubiquitin-protein ligase complex which mediates the ubiquitination and subsequent proteasomal degradation of target proteins. (423 aa) |
| atg7 | Ubiquitin-like modifier-activating enzyme ATG7; E1-like activating enzyme involved in the 2 ubiquitin-like systems required for cytoplasm to vacuole transport (Cvt) and autophagy. Activates ATG12 for its conjugation with ATG5 as well as the ATG8 family proteins for their conjugation with phosphatidylethanolamine. Both systems are needed for the ATG8 association to Cvt vesicles and autophagosomes membranes. Required for autophagic death induced by caspase-8 inhibition. Required for mitophagy which contributes to regulate mitochondrial quantity and quality by eliminating the mitochondria [...] (711 aa) |
| hsp70l | Novel protein similar to heat shock cognate 70-kd protein; Belongs to the heat shock protein 70 family. (643 aa) |
| ywhaqb | Tyrosine 3-monooxygenase/tryptophan 5-monooxygenase activation protein, theta polypeptide b; Belongs to the 14-3-3 family. (245 aa) |
| tlr3 | Toll-like receptor 3; Belongs to the Toll-like receptor family. (903 aa) |
| LOC100333521 | Pept_C1 domain-containing protein; Belongs to the peptidase C1 family. (303 aa) |
| tlr20.3 | Toll-like receptor 20, tandem duplicate 3. (175 aa) |
| traf3 | TNF receptor-associated factor; Belongs to the TNF receptor-associated factor family. (573 aa) |
| ecsit | Evolutionarily conserved signaling intermediate in Toll pathway, mitochondrial; Adapter protein of the Toll-like and IL-1 receptor signaling pathway that is involved in the activation of NF-kappa-B. (452 aa) |
| txnl4b | Thioredoxin-like protein; Plays role in pre-mRNA splicing. Belongs to the DIM1 family. (149 aa) |
| myh7 | Myosin heavy chain 7; Belongs to the TRAFAC class myosin-kinesin ATPase superfamily. Myosin family. (1938 aa) |
| tlr4bb | Toll-like receptor 4b, duplicate b. (819 aa) |
| pak1ip1 | P21-activated protein kinase-interacting protein 1-like; Negatively regulates the PAK1 kinase. PAK1 is a member of the PAK kinase family, which has been shown to play a positive role in the regulation of signaling pathways involving MAPK8 and RELA. PAK1 exists as an inactive homodimer, which is activated by binding of small GTPases such as CDC42 to an N-terminal regulatory domain. PAK1IP1 also binds to the N-terminus of PAK1, and inhibits the specific activation of PAK1 by CDC42. May be involved in ribosomal large subunit assembly. (368 aa) |
| tab3 | Mitogen-activated protein kinase kinase kinase 7 interacting protein 3 like. (573 aa) |
| LOC798445 | Uncharacterized protein; Belongs to the peptidase C14A family. (184 aa) |
| irak4 | Interleukin-1 receptor-associated kinase 4. (447 aa) |
| socs2 | Suppressor of cytokine signaling 2. (197 aa) |
| mak16 | Protein MAK16 homolog; Plays an essential role in early embryonic development. (305 aa) |
| pxdn | Peroxidasin. (1460 aa) |
